# Supplementary material for: Loss of WIPI4 in neurodegeneration causes autophagy-independent ferroptosis
Source: Nat Cell Biol. 2024 Mar 7;26(4):542–51. doi: 10.1038/s41556-024-01373-3 (PMC11021183; doi:10.1038/s41556-024-01373-3)
Supplement: Supplementary file 1 — Reporting Summary [file 41556_2024_1373_MOESM1_ESM.pdf]

nature portfolio

Corresponding author(s): David C Rubinsztein

Last updated by author(s): 23/01/24

Reporting Summary

Nature Portfolio wishes to improve the reproducibility of the work that we publish. This form provides structure for consistency and transparency in reporting. For further information on Nature Portfolio policies, see our Editorial Policies and the Editorial Policy Checklist.

Statistics

For all statistical analyses, confirm that the following items are present in the figure legend, table legend, main text, or Methods section.

n/a	Confirmed	
☐	☒	The exact sample size (*n*) for each experimental group/condition, given as a discrete number and unit of measurement
☐	☒	A statement on whether measurements were taken from distinct samples or whether the same sample was measured repeatedly
☐	☒	The statistical test(s) used AND whether they are one- or two-sided<br>*Only common tests should be described solely by name; describe more complex techniques in the Methods section.*
☒	☐	A description of all covariates tested
☒	☐	A description of any assumptions or corrections, such as tests of normality and adjustment for multiple comparisons
☐	☒	A full description of the statistical parameters including central tendency (e.g. means) or other basic estimates (e.g. regression coefficient) AND variation (e.g. standard deviation) or associated estimates of uncertainty (e.g. confidence intervals)
☐	☒	For null hypothesis testing, the test statistic (e.g. *F*, *t*, *r*) with confidence intervals, effect sizes, degrees of freedom and *P* value noted<br>*Give P values as exact values whenever suitable.*
☒	☐	For Bayesian analysis, information on the choice of priors and Markov chain Monte Carlo settings
☒	☐	For hierarchical and complex designs, identification of the appropriate level for tests and full reporting of outcomes
☐	☒	Estimates of effect sizes (e.g. Cohen's *d*, Pearson's *r*), indicating how they were calculated

Our web collection on statistics for biologists contains articles on many of the points above.

Software and code

Policy information about availability of computer code

Data collection	Confocal images were collected with ZEN Black 2.6 Carl Zeiss Microscopy. Superresolution images were acquired with Zen 20 12 Elyra edition software.<br>Plate reader Tecan SparkControl V3.0.<br>The IncucyteS3 incubated live imaging system.<br>Images of zebrafish cryosections were taken by the Qlmaging Retiga 2000 R digital camera using Qcapture software.<br>Live imaging of zebrafish larvae injected with ATG2A constructs were taken using Leica Application Suite X (LAS X) software<br>Attune'" NxT BD FACSDiva'M Software.
Data analysis	IMAGE STUDIO Lite LI-COR ver 5.2, Inc and Image J (National Institute of Health, USA) for gel analysis.<br>ZEN imaging software (ZEN Black 2.3 Carl Zeiss Microscopy) and Image J ver 1.54f for microscopic image analysis.<br>Volocity 6.3 Software (PerkinElmer) for Pearson's correlation coefficient (PCC).<br>Microsoft Excel (Excel 2016 Microsoft office) and GraphPad Prism v9 (GraphPad Software) for statistical analysis.<br>lmaris image analysis software<br>lncucyte 2020 software<br>FlowJo vl0.8<br>SparkControl V3.0<br>LightCycler® 480 Software (Vl.5.1.62)

For manuscripts utilizing custom algorithms or software that are central to the research but not yet described in published literature, software must be made available to editors and reviewers. We strongly encourage code deposition in a community repository (e.g. GitHub). See the Nature Portfolio guidelines for submitting code & software for further information.

Data

Policy information about availability of data

All manuscripts must include a data availability statement. This statement should provide the following information, where applicable:

- Accession codes, unique identifiers, or web links for publicly available datasets
- A description of any restrictions on data availability
- For clinical datasets or third party data, please ensure that the statement adheres to our policy

All data supporting the findings of this study are available from the corresponding author upon reasonable request. Source data are provided with this paper.

Research involving human participants, their data, or biological material

Policy information about studies with human participants or human data. See also policy information about sex, gender (identity/presentation), and sexual orientation and race, ethnicity and racism.

Reporting on sex and gender	not applicable
Reporting on race, ethnicity, or other socially relevant groupings	not applicable
Population characteristics	not applicable
Recruitment	not applicable
Ethics oversight	not applicable

Note that full information on the approval of the study protocol must also be provided in the manuscript.

Field-specific reporting

Please select the one below that is the best fit for your research. If you are not sure, read the appropriate sections before making your selection.

☒ Life sciences ☐ Behavioural & social sciences ☐ Ecological, evolutionary & environmental sciences

For a reference copy of the document with all sections, see nature.com/documents/nr-reporting-summary-flat.pdf

Life sciences study design

All studies must disclose on these points even when the disclosure is negative.

Sample size	Sample sizes were chosen on the basis of extensive experience with the assays we have performed. (e.g. Wrobel et al. Nature Communications 2022)
Data exclusions	No data were excluded from the analysis.
Replication	Mammalian: Cell death, viability, MDA assays, and western blots, usually 3 biological repeats were done for each experiment. For immunofluorescence experiments, 5-50 images were taken for each biological repeat and 3 biological repeats were done for each experiment. Zebrafish: For the analysis of images of fish retinal degeneration a minimum of 12 eyes per group were quantified. For the phenotypic analysis of fish after ATG2A, a minimum of 4 set of injections for each condition was analysed. For MDA analysis, sample size corresponds to 5 pools of 10 fish each per condition. Expression was analysed in 7 samples (10 fish each) per condition. Survival analysis was done using 60 fish per group. Each replicate was perform at different time.
Randomization	All in vivo experiments and tests were randomly assigned, but no randomization was performed for cell culture experiments.
Blinding	Immunofluorescence analysis was blinded when possible. Western blot analysis was not blinded as it was not possible as the gel loading order needs to be defined. Investigators were not blinded during the other experiments.

Reporting for specific materials, systems and methods

We require information from authors about some types of materials, experimental systems and methods used in many studies. Here, indicate whether each material, system or method listed is relevant to your study. If you are not sure if a list item applies to your research, read the appropriate section before selecting a response.

Materials & experimental systems

n/a	Involved in the study	
☐	☒	Antibodies
☐	☒	Eukaryotic cell lines
☒	☐	Palaeontology and archaeology
☐	☒	Animals and other organisms
☒	☐	Clinical data
☒	☐	Dual use research of concern
☒	☐	Plants

Methods

n/a	Involved in the study	
☒	☐	ChIP-seq
☐	☒	Flow cytometry
☒	☐	MRI-based neuroimaging

Antibodies

Antibodies used

Primary antibodies:
Antibodies for western blots:
mouse anti-Flag [M2] (#F3165, RRID:AB_262044, WB 1:2000),
rabbit anti-WIPl4(Cat# 19194-1-AP) from Proteintech, RRID:AB_2215404
mouse anti-α-Tubulin [DM1A] (Cat# T9026) from Sigma-Aldrich, RRID:AB_477593
rabbit anti-actin (#A2066) from Sigma Aldrich, RRID:AB_476693
rabbit anti-ATG2A (PD041) from MBL Life Science, RRID:AB_2810871
rabbit anti-GFP(ab6556) from Abcam, RRID:AB_305564
mouse anti-NDUFA9 [20C11B11B11] (ab14713) from Abcam, RRID:AB_301431
rabbit anti-Sec23a (ab137583) from Abcam
mouse anti-Tom40 [D-2] (sc-365467) from Santa Cruz Biotechnology, RRID:AB_10847086
rabbit anti-TMEM41b (NBPl-81552) from Novus Biologicals, RRID:AB_11015584
rabbit anti-Calreticulin (#12238) from Cell Signalling, RRID:AB_2688013
rabbit anti-cleaved Caspase-3 (#9661) from Cell Signalling, RRID:AB_2341188
rabbit anti-ATG16L1 (#8089) from Cell Signalling, RRID:AB_10950320
rabbit anti-ATG7 (#2631) from Cell Signalling, RRID:AB_2227783
rabbit anti-TOMM20 (ab186735) from Abcam, RRID:AB_2889972
mouse anti-GM130 [EP892Y] (ab52649) from Abcam, RRID:AB_880266
rabbit anti-LAMP1 (ab24170) from Abcam, RRID:AB_775978
rabbit anti-KDEL (ab2898) from Abcam, RRID:AB_303392
rabbit anti-PISD (HPA031091), from Atlas antibodies, RRID:AB_10600893
mouse anti-beta Ill Tubulin antibody [2G10] (ab78078) from Abcam, RRID:AB_2256751
rabbit anti-MAP2 antibody (8707S), from Cell signalling, RRID:AB_10693782
rabbit anti-NIX (12396), from cell signalling, RRID:AB_2688036
mouse anti-ORP8 [PL-C26] (sc-134409), from Santa Cruz, RRID:AB_2156227
rabbit anti-ORP5 (HPA038712), from Atlas Antibodies, RRID:AB_10675949
rabbit anti IP3 Receptor 1 (D53A5)(#8568), from Cell signalling, RRID:AB_10890699
mouse anti VDAC1 [20B12AF2] (ab14734), from Abcam, RRID:AB_443084
Antibodies for immunofluorescence:
mouse anti-TOMM20 F-10 (sc-17764) from Santa Cruz, RRID:AB_628381
mouse anti-Calnexin [6F12BE10] (ab112995), from Abcam, RRID:AB_10860712

Secondary Antibodies: anti-mouse (#NA931V, RRID:AB_772210) and anti-rabbit (#NA934V) horseradish peroxidise (HRP)-conjugated secondary antibodies (GE Healthcare); anti-goat horseradish peroxidise (HRP)-conjugated secondary antibody (#611620, RRID:AB_87867, Invitrogen/Life Technologies). For immunoflourescence, goat-anti-mouse Alexa Fluor 488 (#A11029, RRID:AB_2534088, 1:400), 555 (#A21147, RRID:AB_1500897, 1:400) and 594 (#A11032, RRID:AB_2534091, 1:400), goat-anti-rabbit Alexa Fluor 488 (#A32731, RRID:AB_2633280, 1:400) and 555 (#A21428, RRID:AB_141784, 1:400) from ThermoFisher Scientific.

Validation

All antibodies used in this study were purchased from commercial vendors who had validated specificity in human cells/ mouse tissues for the specific assays (Western blot, immunoprecipitation and/or immunofluorescence). It is described on data sheets and online.
rabbit anti-Sec23a (ab137583) from Abcam was also validated in Lassalle Set al., Oncotarget, 2016. PMC: 27036030

Eukaryotic cell lines

Policy information about cell lines and Sex and Gender in Research

Cell line source(s)

Human cervical epithelium HeLa (ATCC; #CCL-2; CVCL_0030), human neuroblastoma SH-SY5Y (ECACC; #94030304), human embryonic kidney cell line HEK293 (ECACC; #85120602)
CRISPR/Cas9 ATG2A/B double knockout cell line, from Mizushima's lab
The human iPS WT line (KOLF-2) was generated by the Sanger Wellcome Institute Induced Pluripotent Stem Cell Initiative (HipsSci).
ATG16L1 knockout HeLa and its control were made in house.
Beclin 1 knockout HeLa and its control, from Wensheng Wei's lab in Beijing

Authentication

The cell lines were ordered from ATCC, Horizon or Coriell Institute with authentication.

Authentication	HeLa authenticatd by ATCC (by Short Tandem Repeat (STR) profiling; FTA barcode:STRA1466)<br>SH-SY5Y authenticatd by LGC (STR profiling, FTA barcode:STRA1440)<br>HEK293 authenticatd by LGC (STR profiling, FTA barcode:STRA1472)<br>CRISPR/Cas9 ATG2A/B double knockout cell line (Tamura, N. et al. EBS Lett, 2017)<br>The information about the human iPS WT line including the Certificate of Analysis can be found on the website (www.hipsci.org).<br>ATG16L1 knockout HeLa and its control (Bento, C. F. et al., Nature communication,2016)<br>Beclin1 knockout and its control (He, R. et al, Autophagy, 2015)
Mycoplasma contamination	The cells were regularly tested using EZ-PCR Mycoplasma Test Kit (Biological Industries; cat#20-700-20) and Mycrostripl00 (InvivoGen- rep-mys-100). Cells used in this study were mycoplasma negative.
Commonly misidentified lines<br>(See ICLAC register)	no commonly misidentified cell lines were used in the study.

Animals and other research organisms

Policy information about studies involving animals; ARRIVE guidelines recommended for reporting animal research, and Sex and Gender in Research

Laboratory animals	Transgenic Zebrafish line (from Zebrafish Information Network (ZFIN)): Tg2(rho:EGFP)cu3, RRID: ZFIN_ZDB-ALT-101103-1 Wiltype TL.<br>Adult fish of between 6 months and 18 months old were bred to generate embryos and larvae for the experiments described below.<br>Zebrafish from 0 - 7 weeks old were used for Fig 2a (survival assay, Protocol 7).<br>Zebrafish larvae from 0 - 10 days post-fertilisation (10 d.p.f.) were used for Fig 2 b& c.<br>Zebrafish larvae from 0 -5 d.p.f. were used for Fig 2d.<br>Zebrafish larvae from 0 - 10 d.p.f. were used for Fig 3c& d.<br>Zebrafish larvae at 5 d.p.f. were used for Extended Fig 1l.<br>Zebrafish larvae from 0 - 10 d.p.f. were used for Extended Fig 1m.<br>Zebrafish larvae from 0 - 2 d.p.f. were used for Fig 6i, Extended Fig 8b.
Wild animals	No wild animals were used in the study.
Reporting on sex	For zebrafish sex cannot be differented at the stages experiments were done.
Field-collected samples	No field collected samples were used in the study.
Ethics oversight	All zebrafish experiments were performed in accordance with the UK Animals (Scientific Procedures) Act with appropriate Home Office Project and Personal animal licenses and with local Ethics Committee approval. Studies were performed in accordance with PREPARE and ARRIVE guidelines.

Note that full information on the approval of the study protocol must also be provided in the manuscript.

Flow Cytometry

Plots

Confirm that:

- [x] The axis labels state the marker and fluorochrome used (e.g. CD4-FITC).
- [x] The axis scales are clearly visible. Include numbers along axes only for bottom left plot of group (a 'group' is an analysis of identical markers).
- [ ] All plots are contour plots with outliers or pseudocolor plots.
- [x] A numerical value for number of cells or percentage (with statistics) is provided.

Methodology

Sample preparation	The cell line used for BPC11 staining is SH-SY5Y cells detailed in above sections. Cells were stained for 30 minutes in growth media supplemented with 5 µM C11-Bodipy 581/591. All cells in the media and lifted are subject to analysis. The cell line used for NAO staining was HeLa.
Instrument	Becton Dickinson LSR Fortessa
Software	FLowJo TM Vl0.8
Cell population abundance	At least 50000 cells were analysed for each sample.
Gating strategy	For BPC11 staining, first, SH-SY5Y cells were isolated using forward scatter (FSC) and side scatter (SSC) properties. Live cells were distinguished from debris that are of lower FSC and SSC. Then, single cell population was defined by comparing the area of forward scatter (FSC-A) to the height of forward scatter (FSC-H). Cells in which FSC-A is not correlated with FSC-H are likely to be doublets and were therefore excluded from analysis. Last, SH-SY5Y cells positive of BodipyCll staining was defined by thresholding against unstained SH-SY5Y cells. Cells with greater red (YLl-A) fluorescence than that of unstained cells (YLl-A+) were selected as positive of BodipyCll staining (Q2 and Q3). The autofluorescnce of cells was also gated out by thresholding

against unstained SH-SY5Y cells. Cells in Q2 are positive of both reduced and oxidised BodipyC11 signal and are used for quantitative analysis. For NAO staining, median fluorescence intensity analysis of labelled mitochondria was performed by gating on single cells.

☒ Tick this box to confirm that a figure exemplifying the gating strategy is provided in the Supplementary Information.
