# Supplementary material for: Loss of WIPI4 in neurodegeneration causes autophagy-independent ferroptosis
Source: Nat Cell Biol. 2024 Mar 7;26(4):542–51. doi: 10.1038/s41556-024-01373-3 (PMC11021183; doi:10.1038/s41556-024-01373-3)
Supplement: Supplementary file 5 — Uncropped blots. [file 41556_2024_1373_MOESM5_ESM.pdf]

Figure 4b WB fractionation WIPI4 KD for ATG2 in HeLa

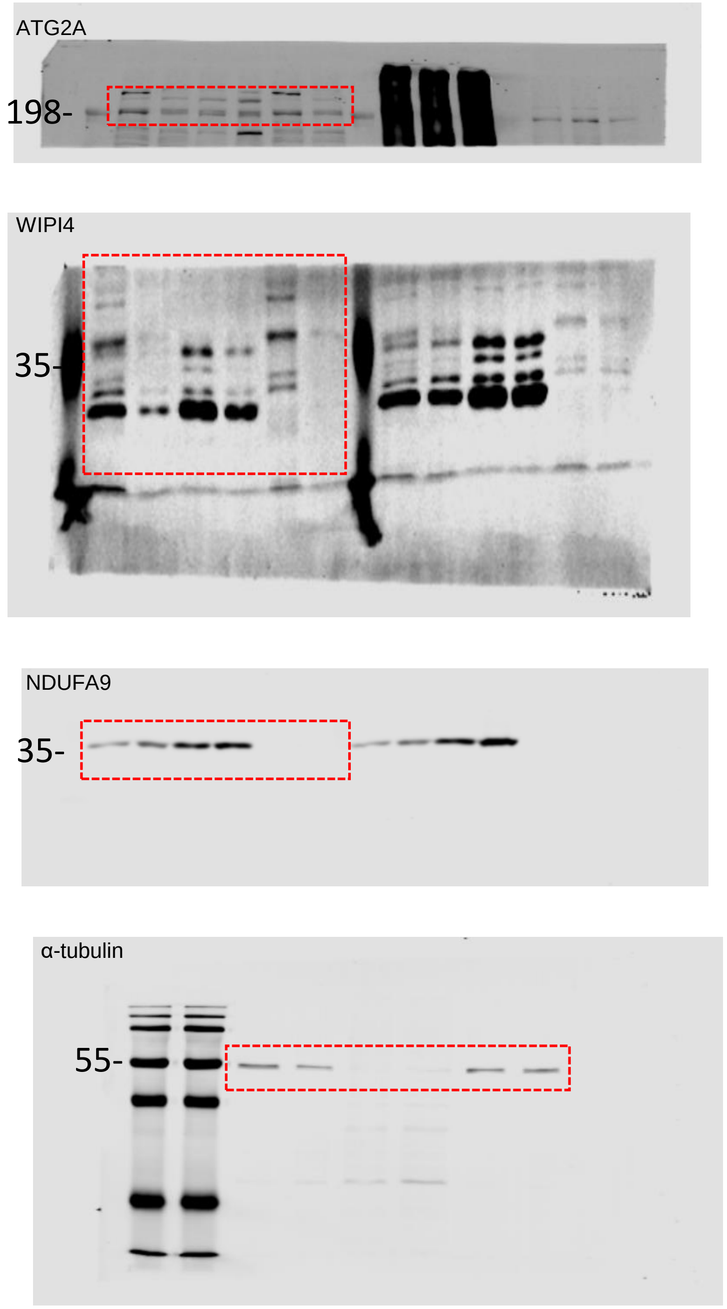

Figure 4d WB IP Interaction TMEM41 and Tom40 through ATG2 in HeLa

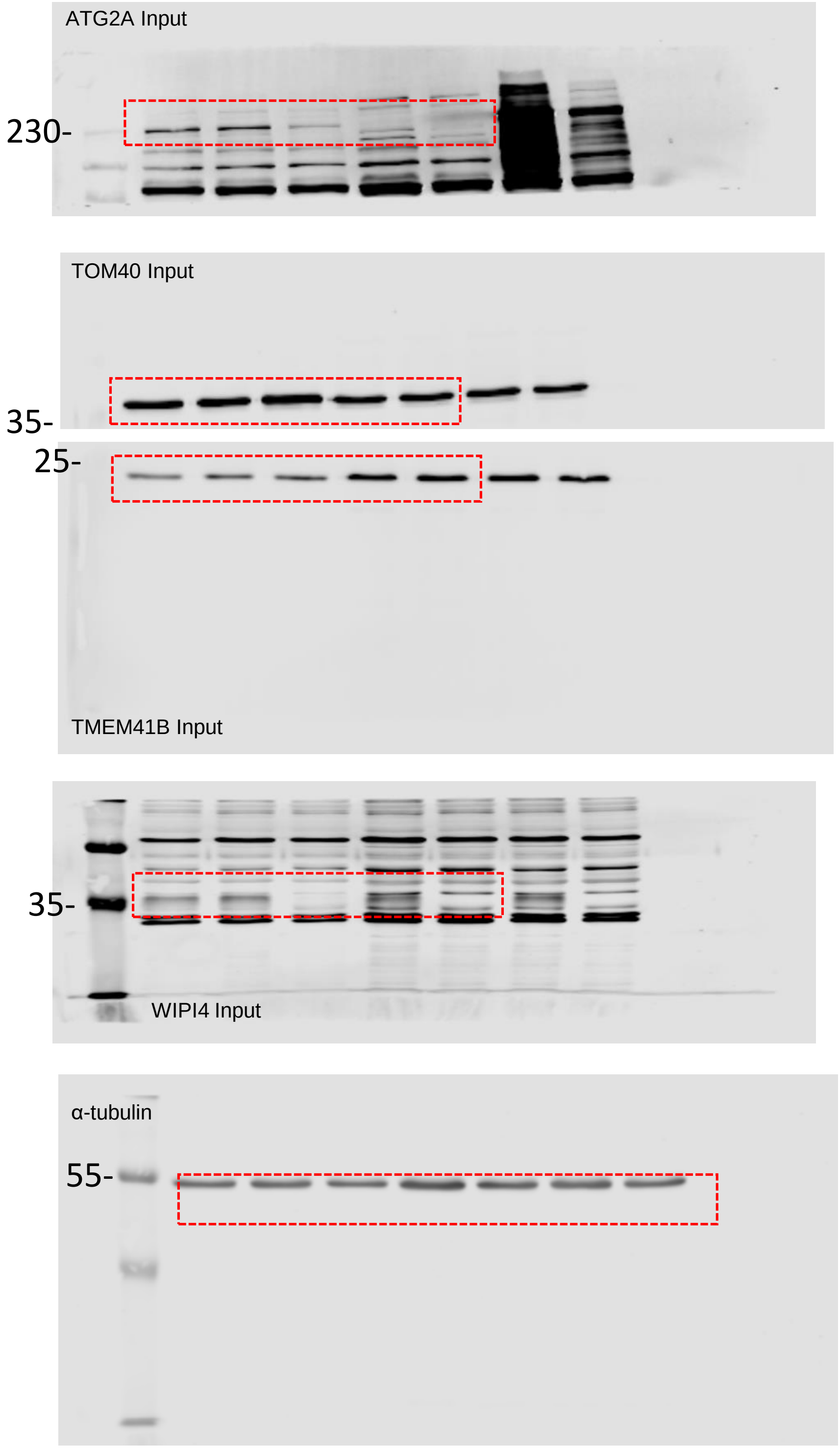

Figure 4d WB IP Interaction TMEM41 and Tom40 through ATG2 in HeLa

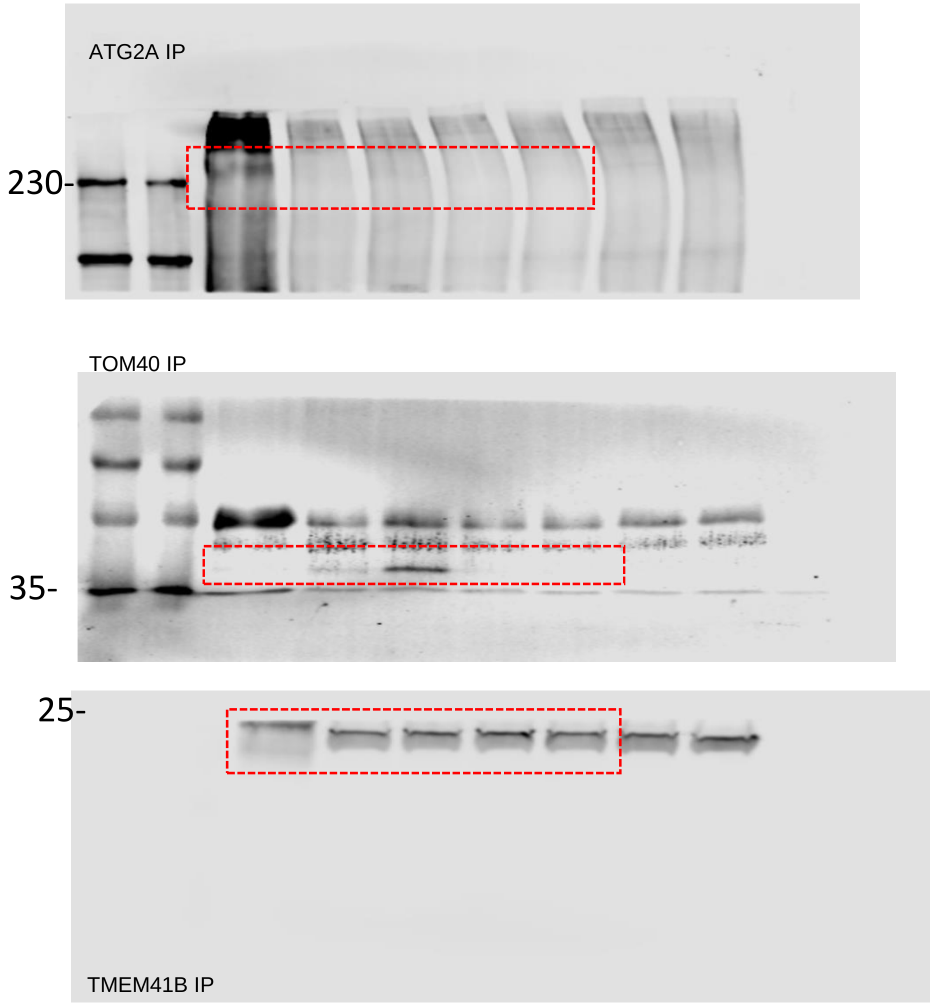

Figure 5a TOMM 40 and WIPI4 KD

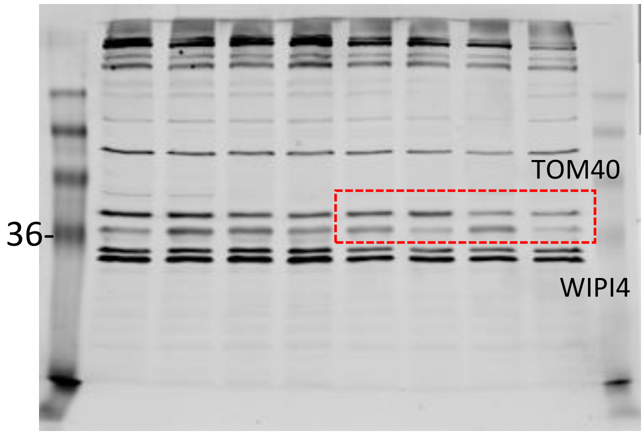

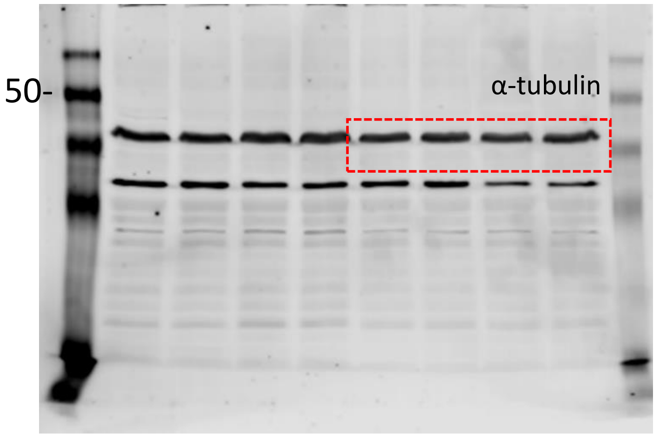

Figure 5c ATG2A mutant fractionation

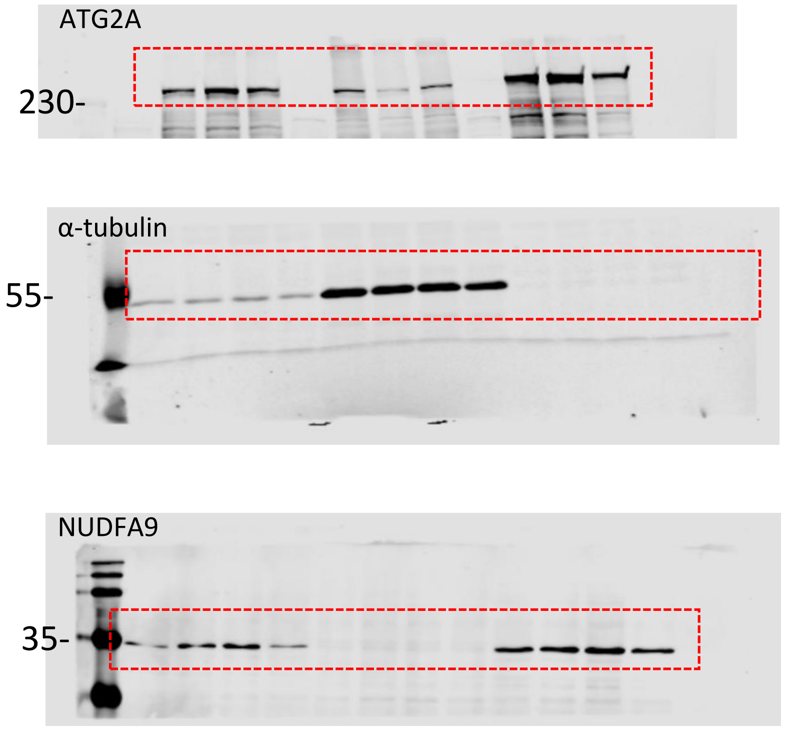

Figure 5d WB fractionation ATG2 in ATG2 KO cells

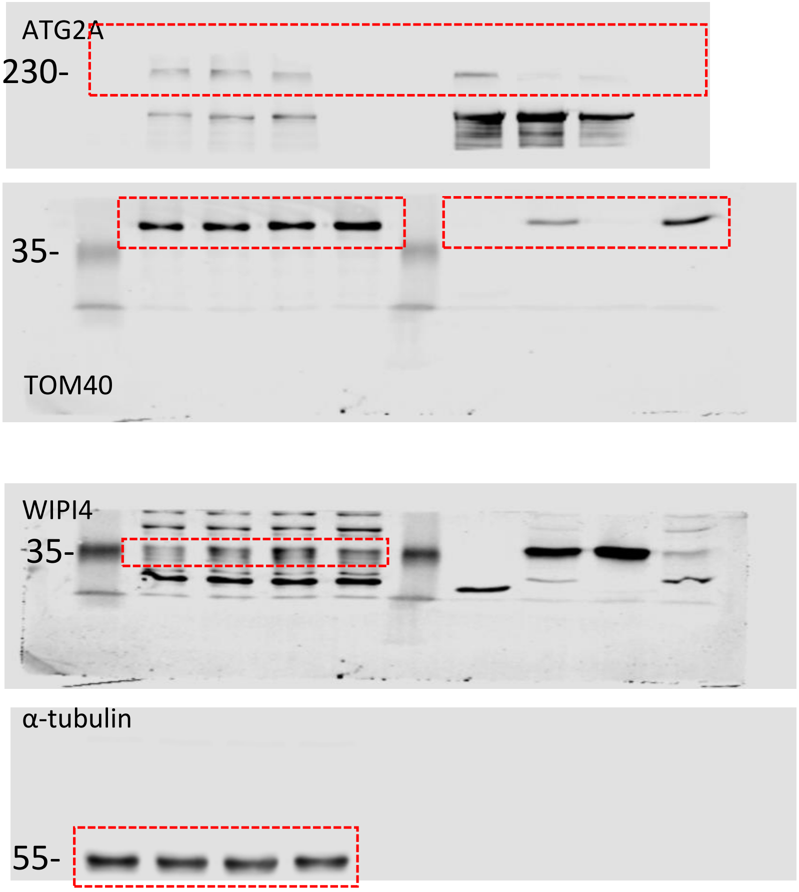

Figure S1a WIPI4 KD efficiency

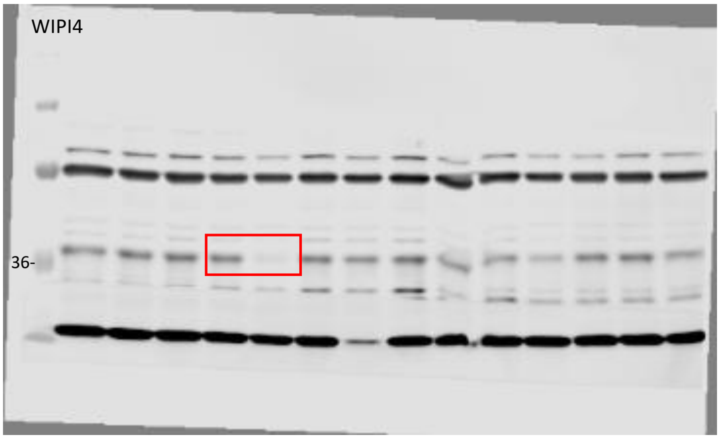

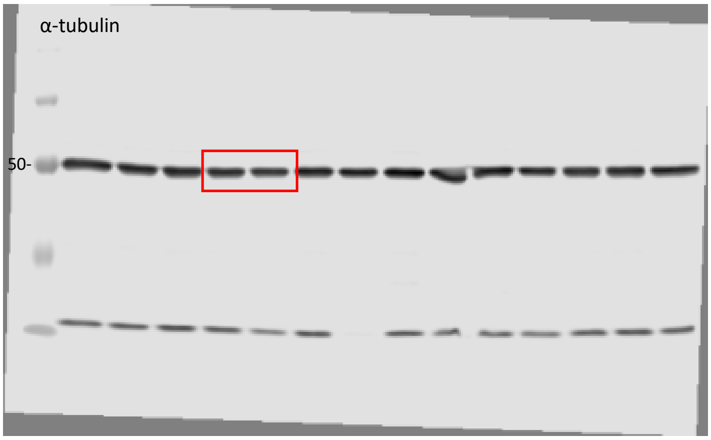

Figure S1b WIPI4 KD efficiency

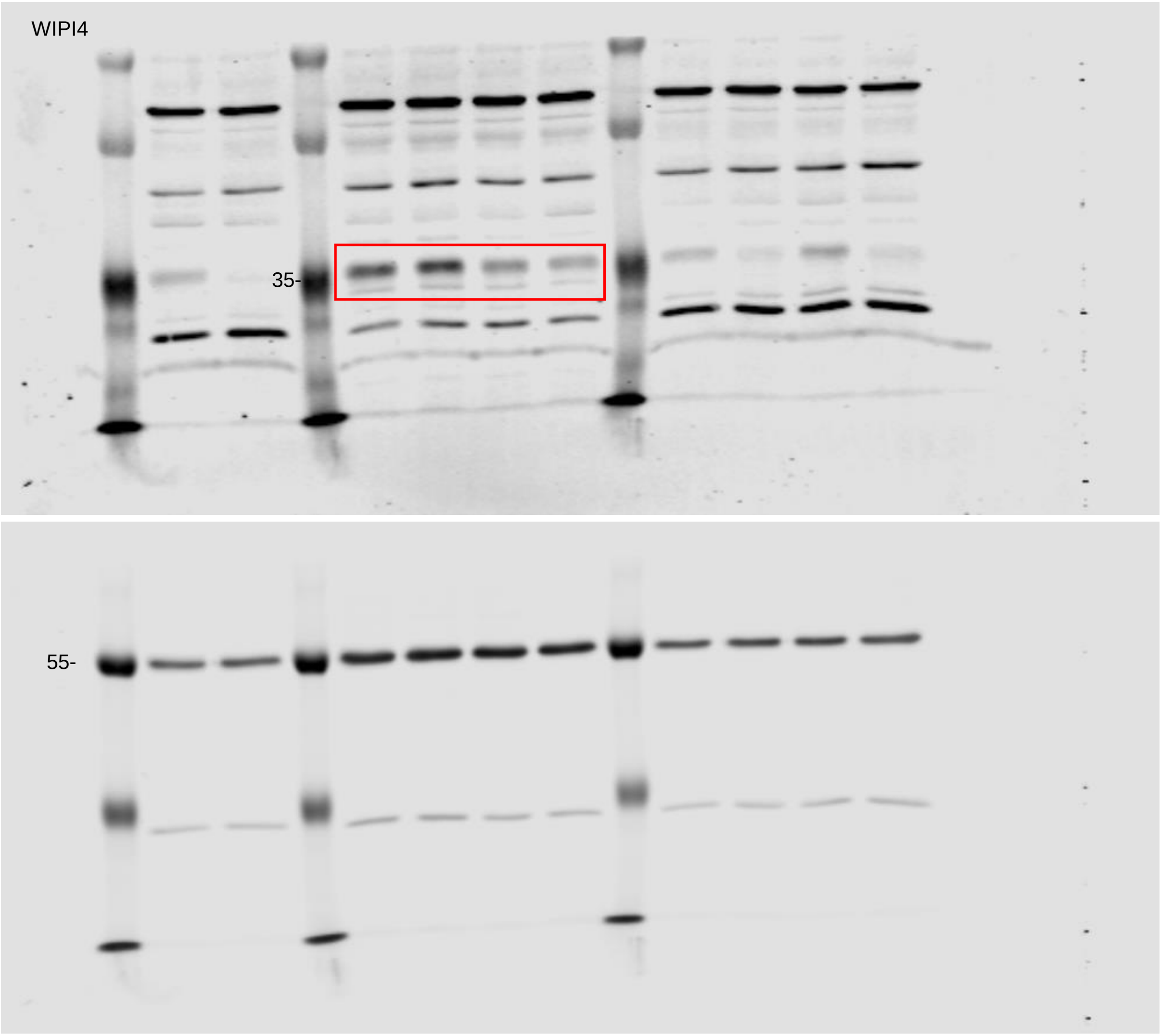

Figure S1c WIPI4 KD efficiency

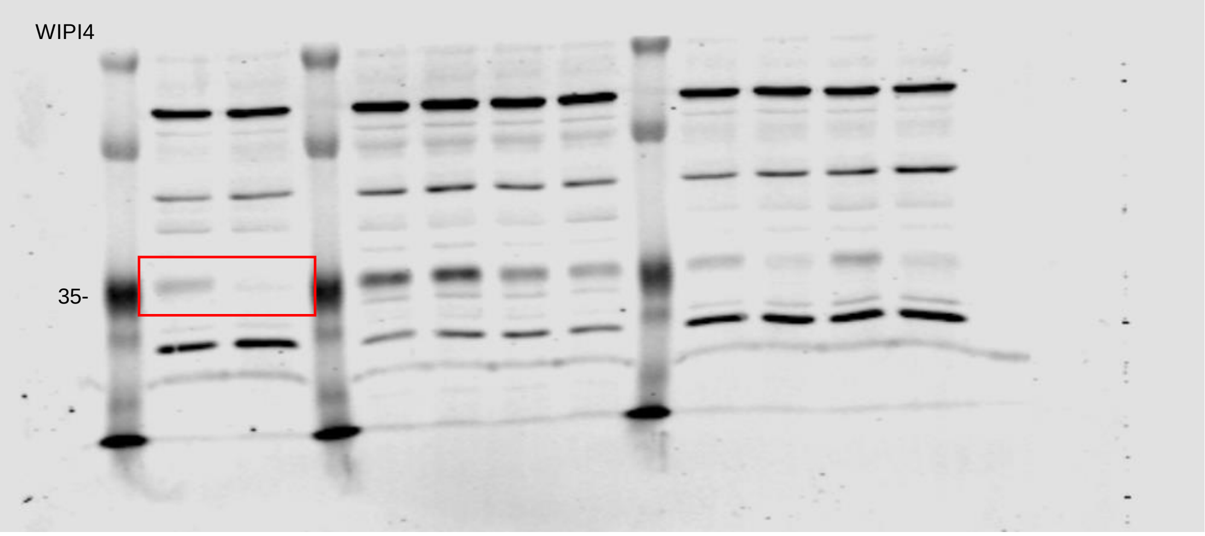

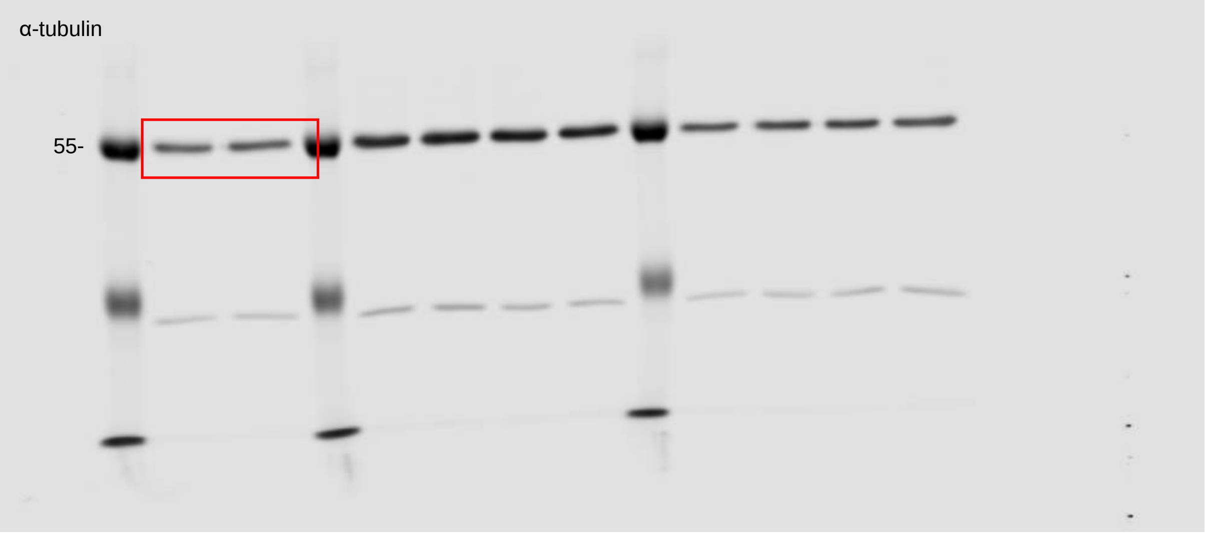

Figure S1d WIPI4 KD efficiency

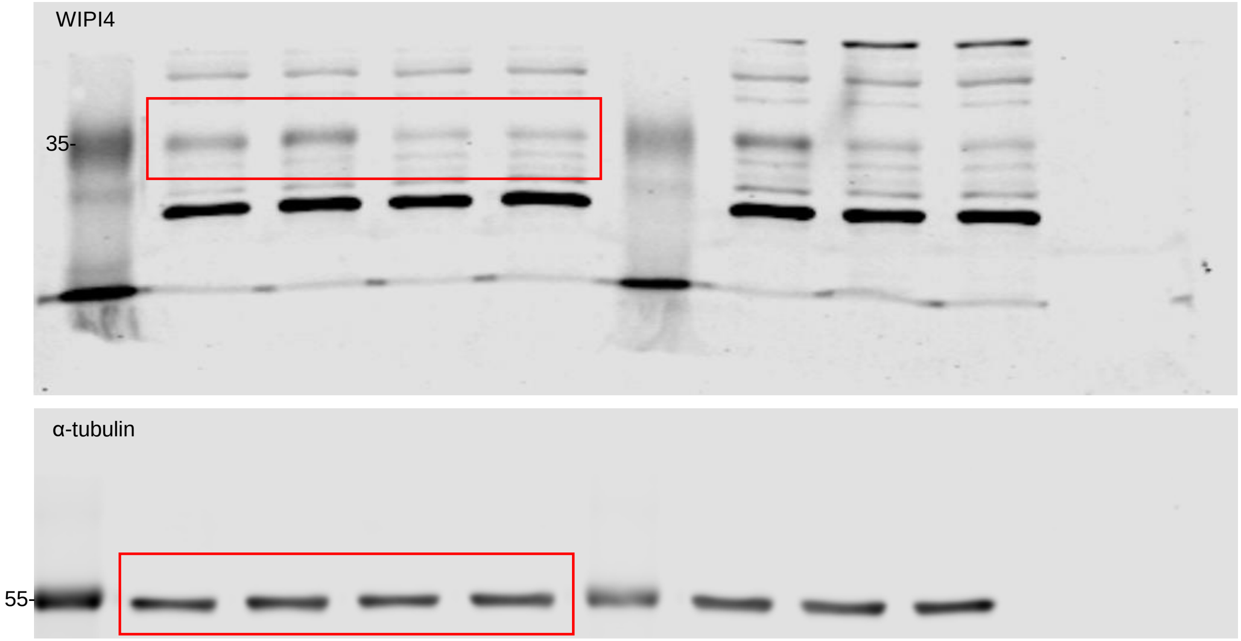

Figure S1e Caspase 3 levels

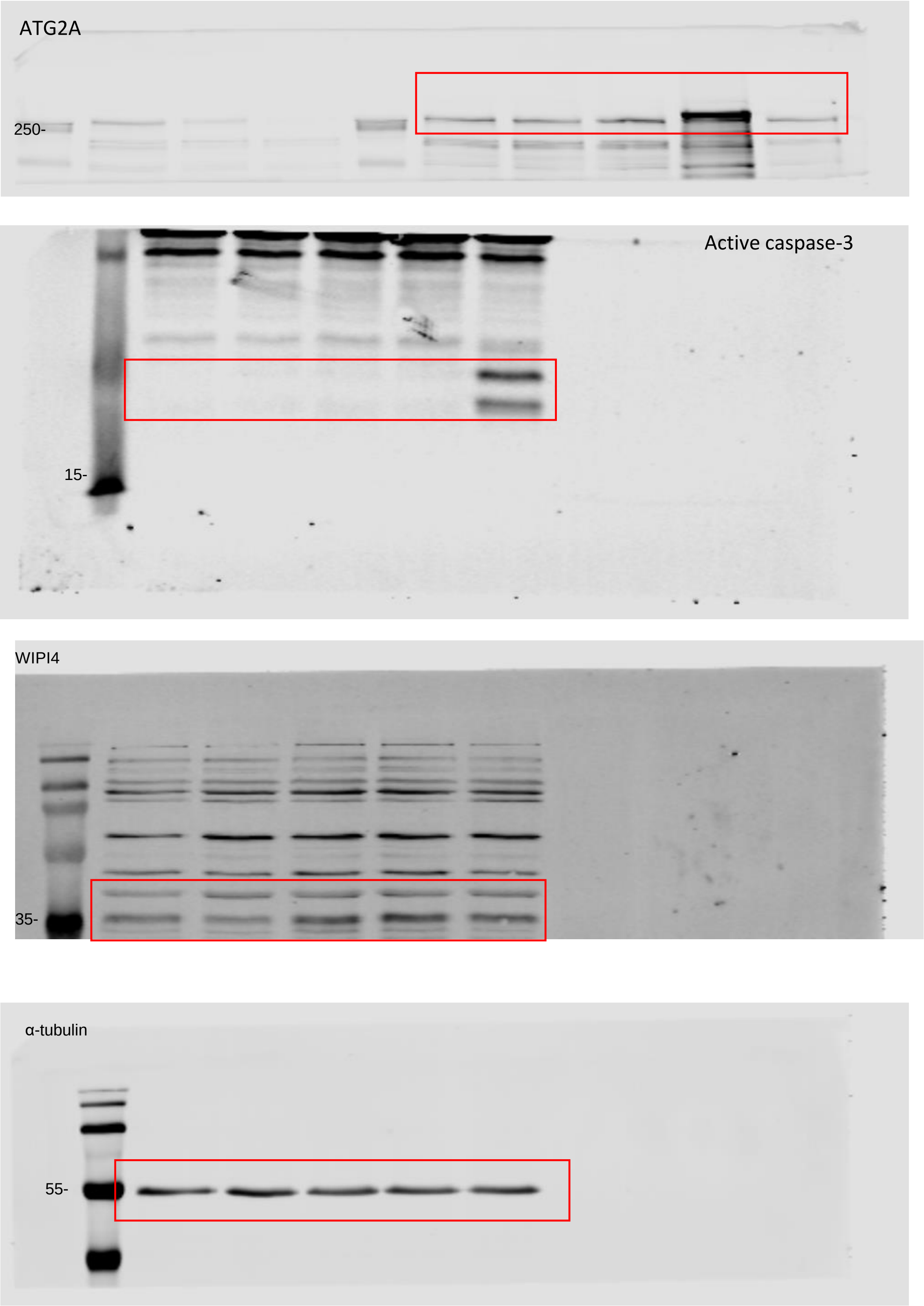

Figure S1f CRISPR KO efficiency of WIPI4 over time

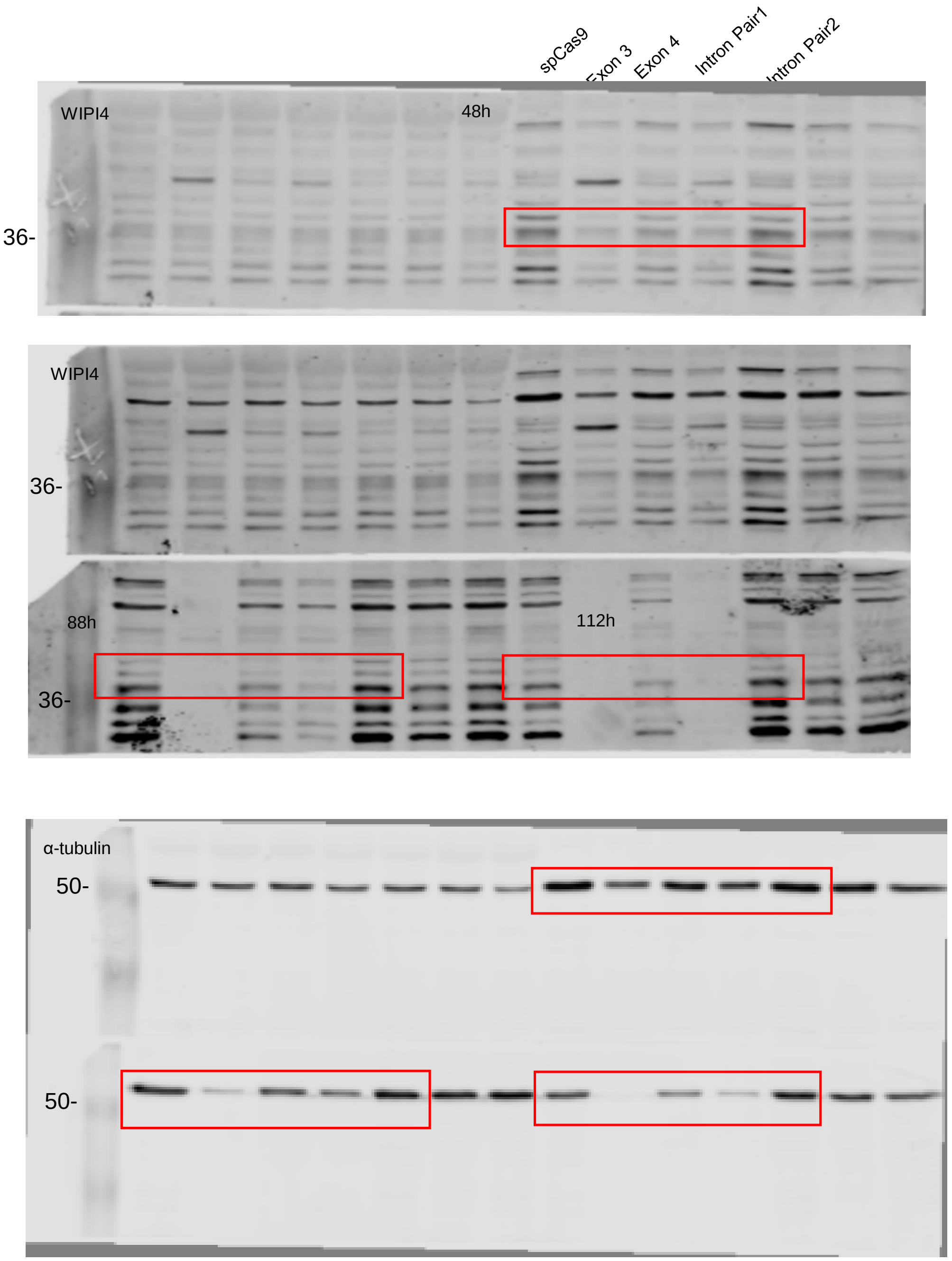

Figure S1j WIPI4 KD efficiency by shRNA in iPSC neurons

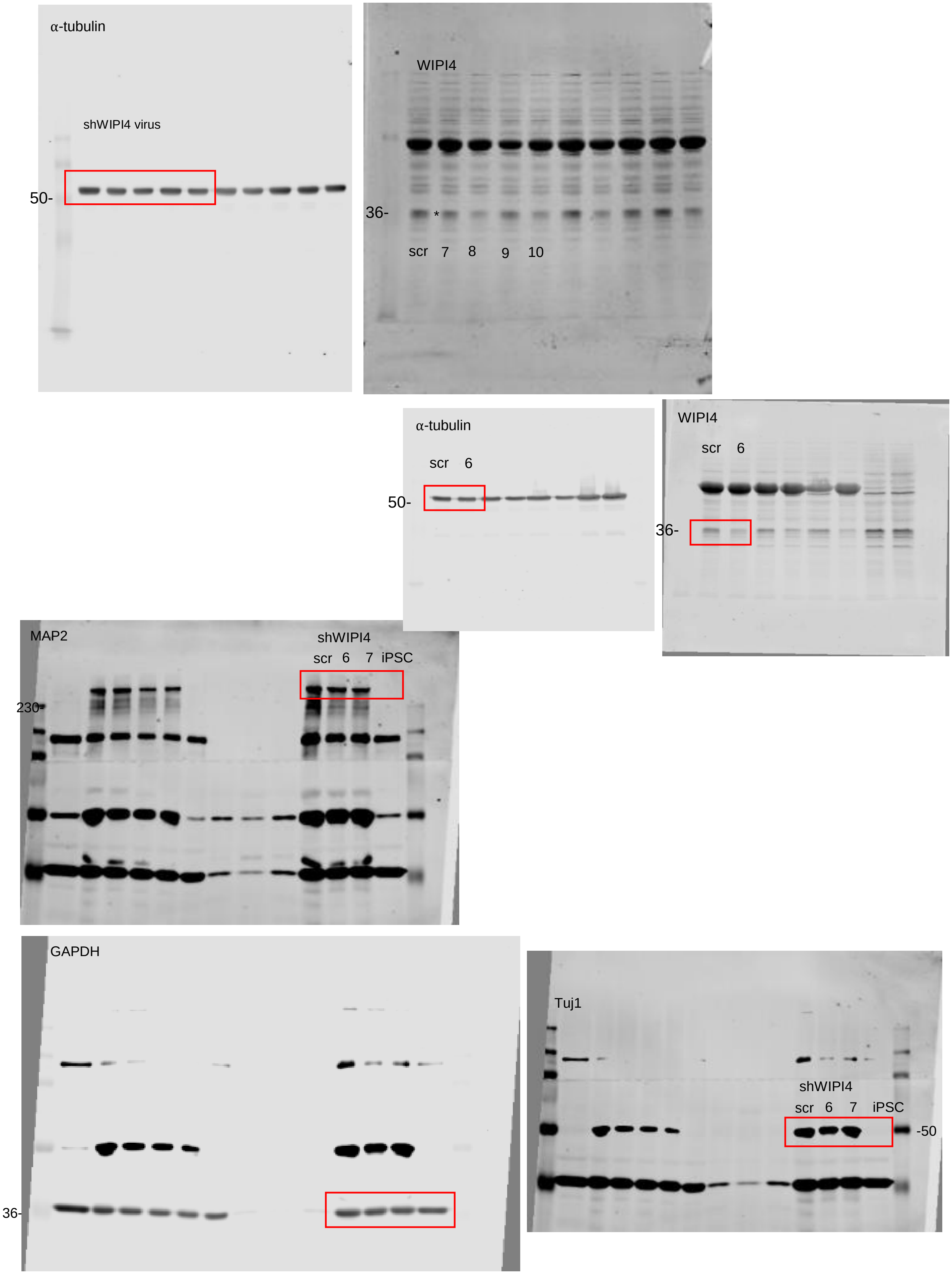

Figure S1k WIPI4 KD efficiency in mouse primary neurons

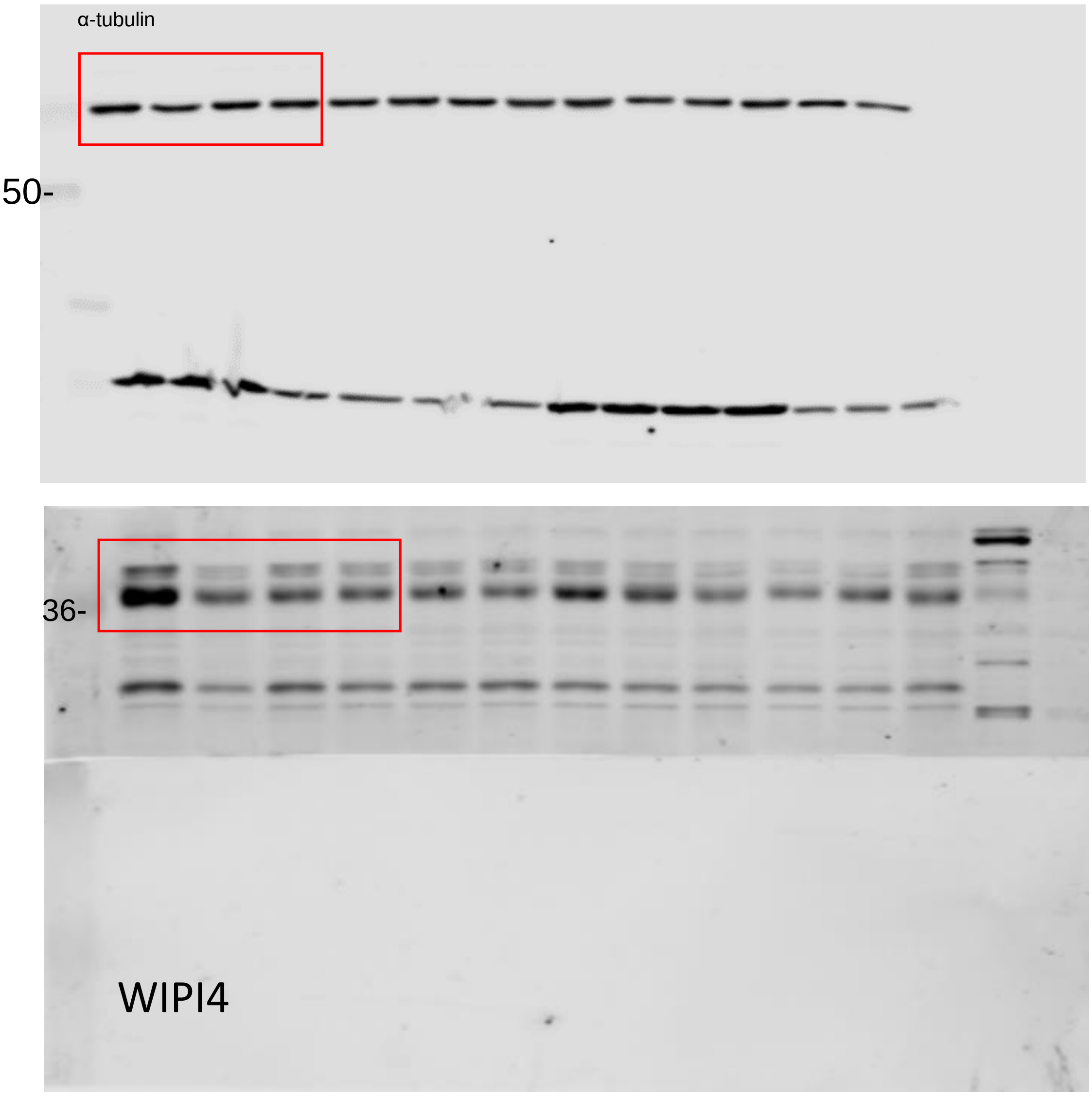

Figure S2a WIPI2 KD efficiency

WIPI2

55 kDa-

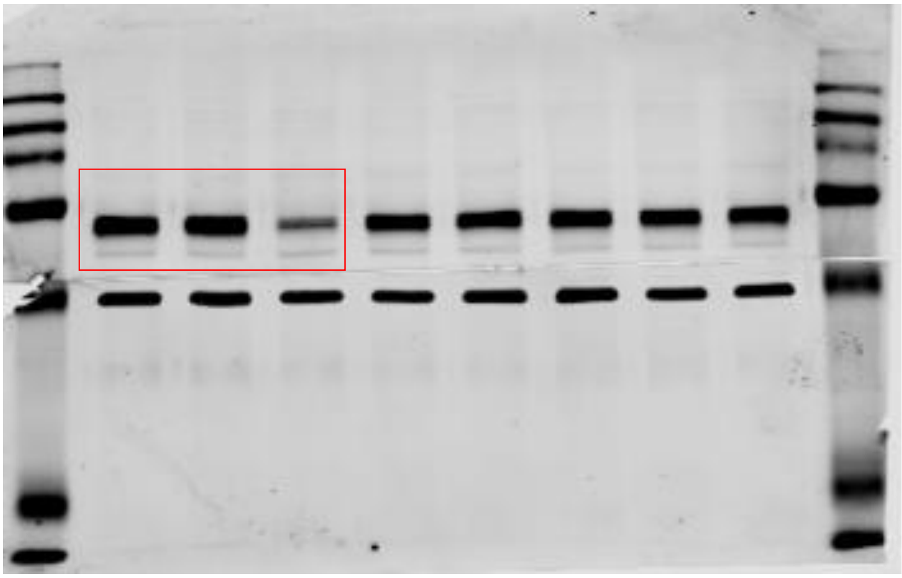

55 kDa-

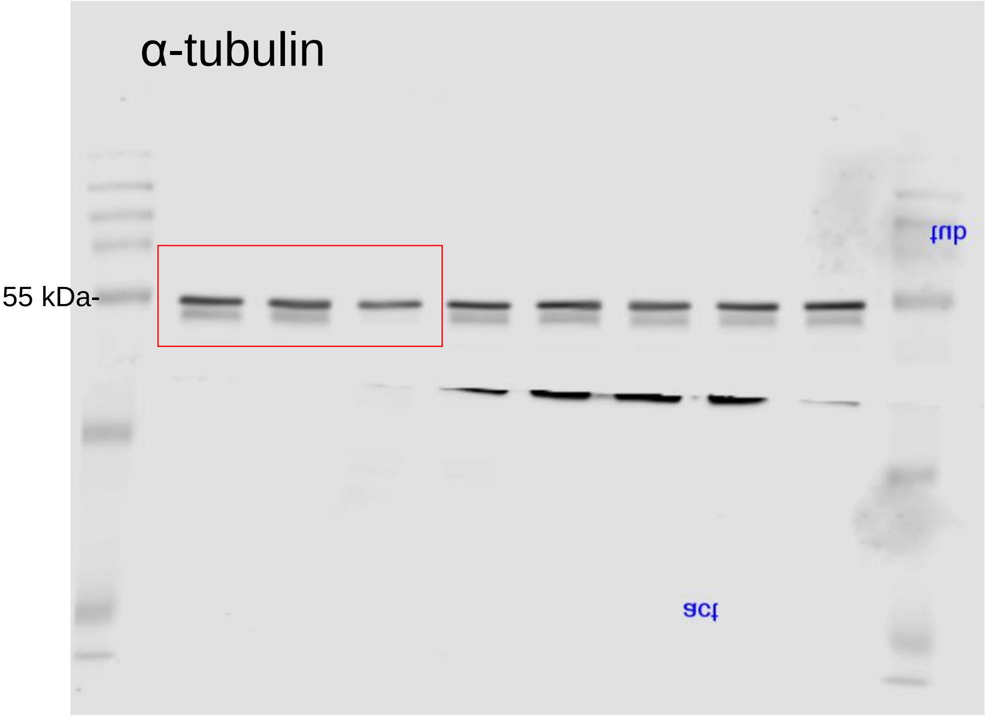

Figure S2b KD efficiency of ATG proteins

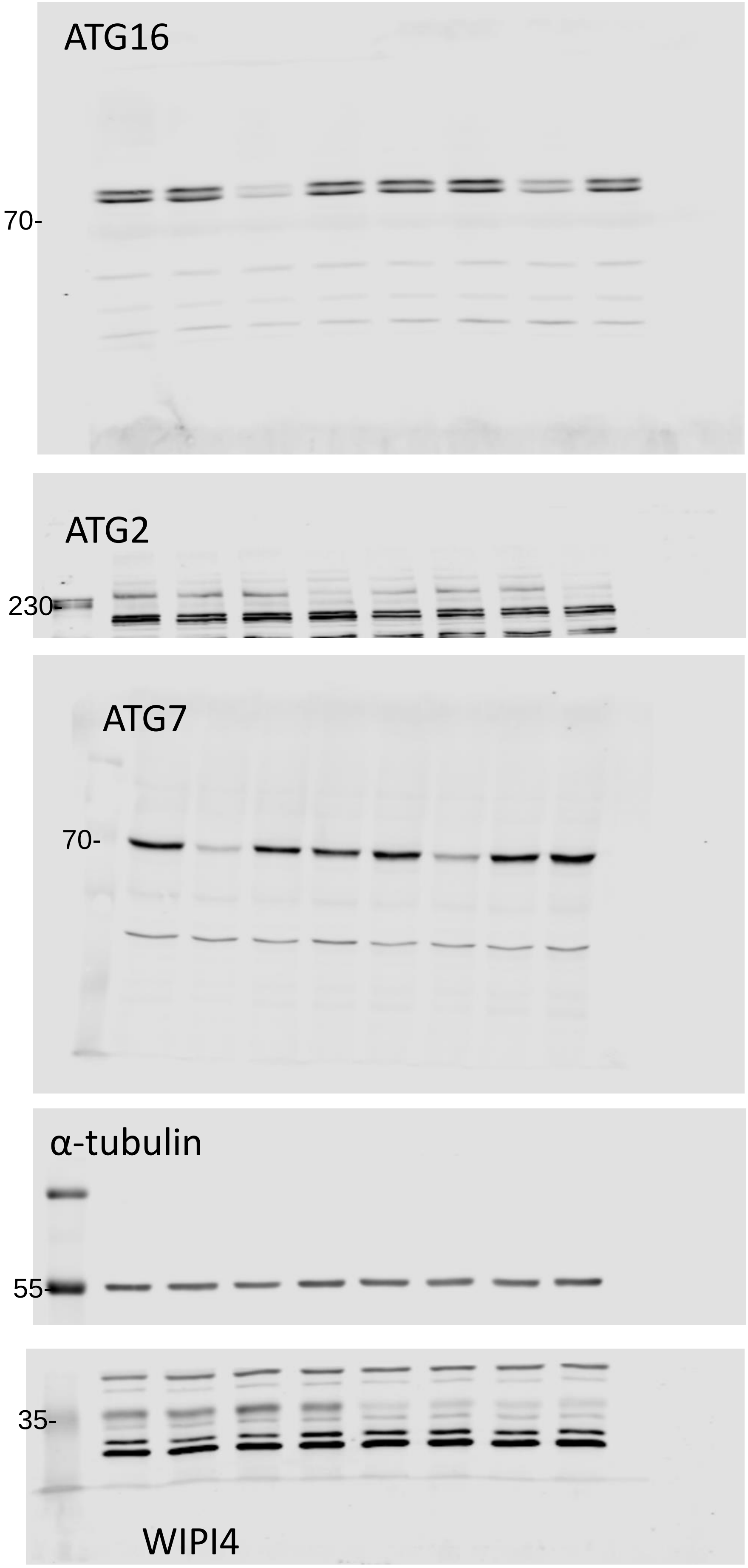

Figure S2d ATG16 KO HeLa cells do not have LC3 conjugation

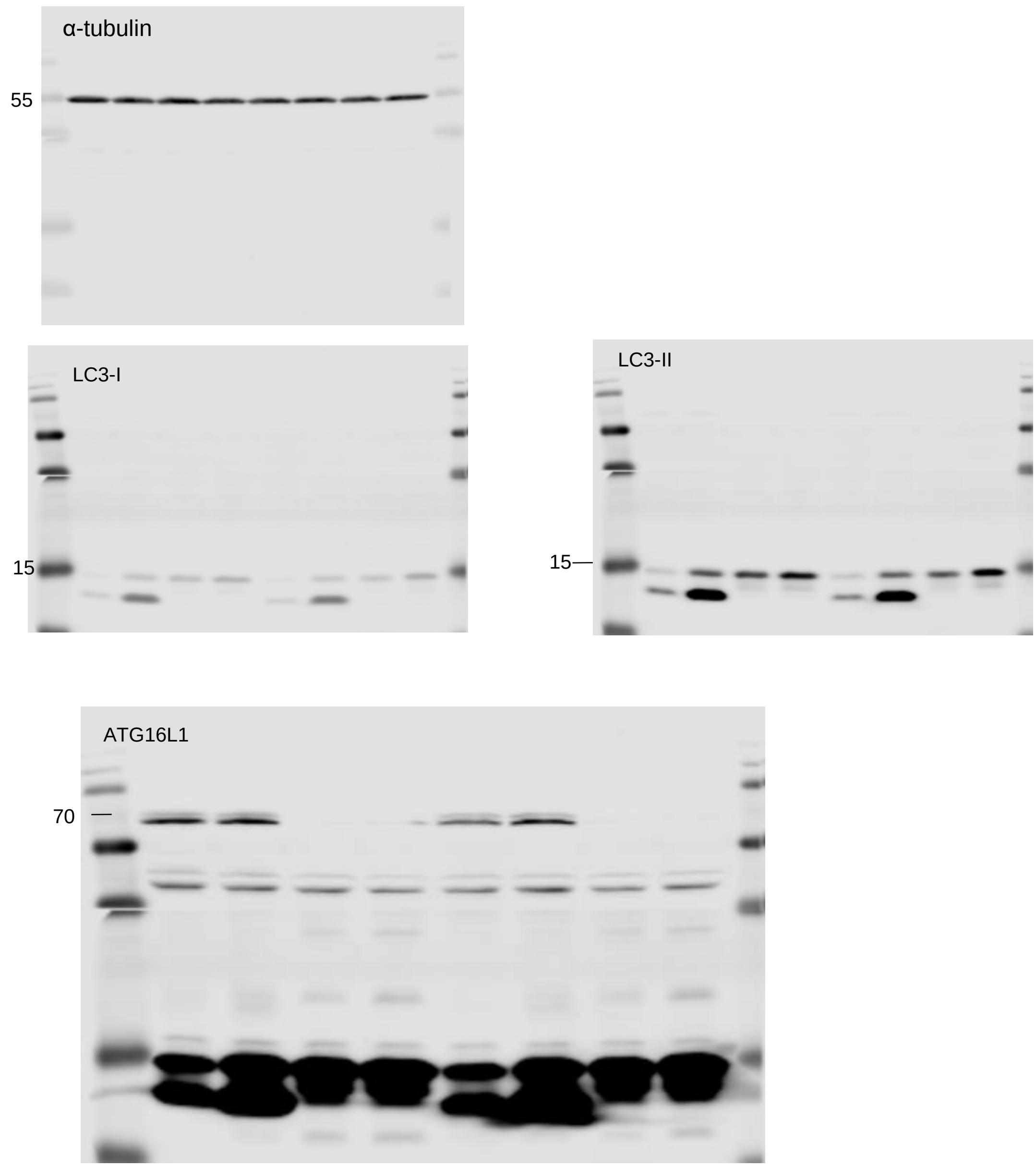

Figure S2e siWIPI4 in BECN1 KO cells

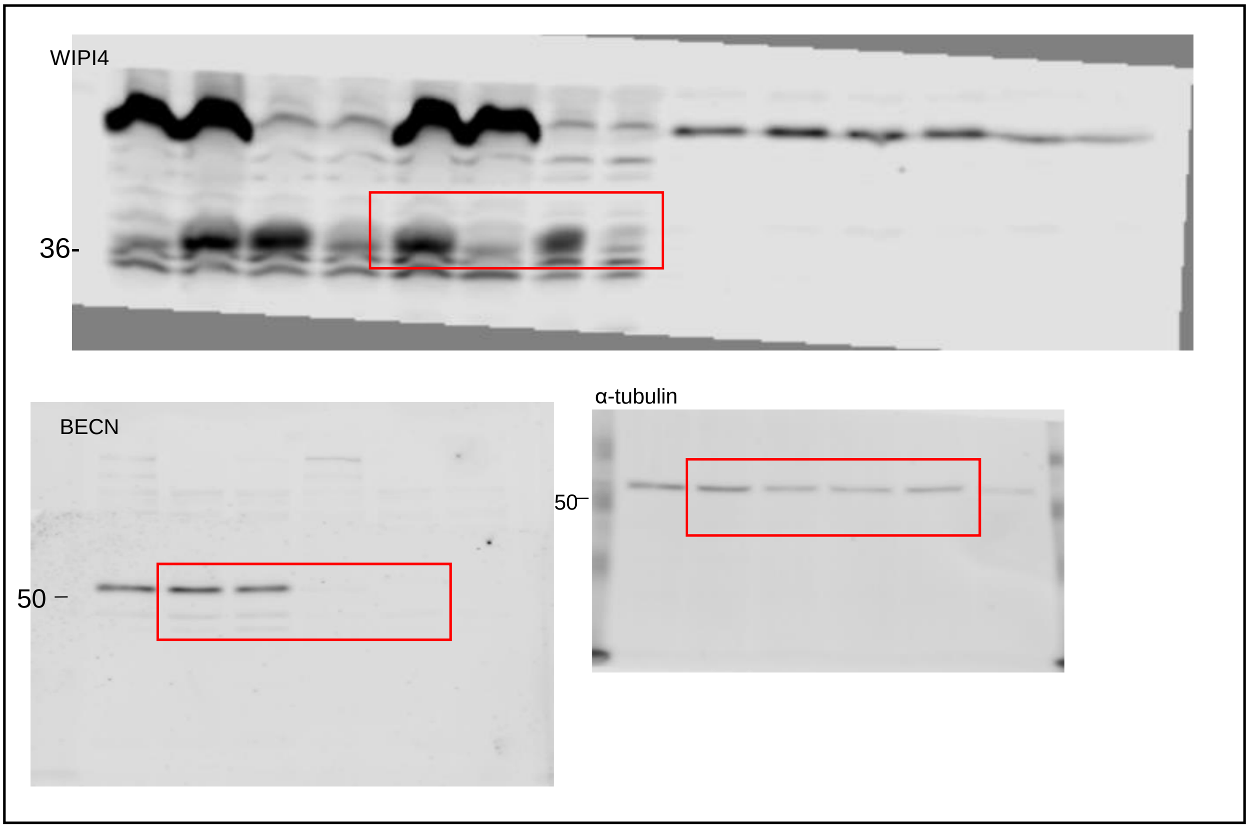

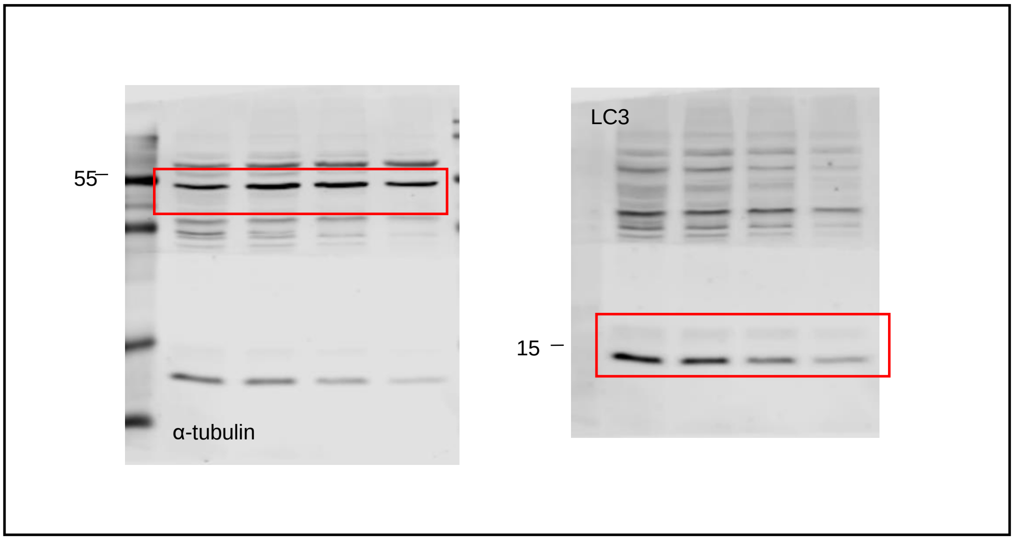

Figure S2f siWIPI4 in ULK1 inhibitor treated HeLa cells

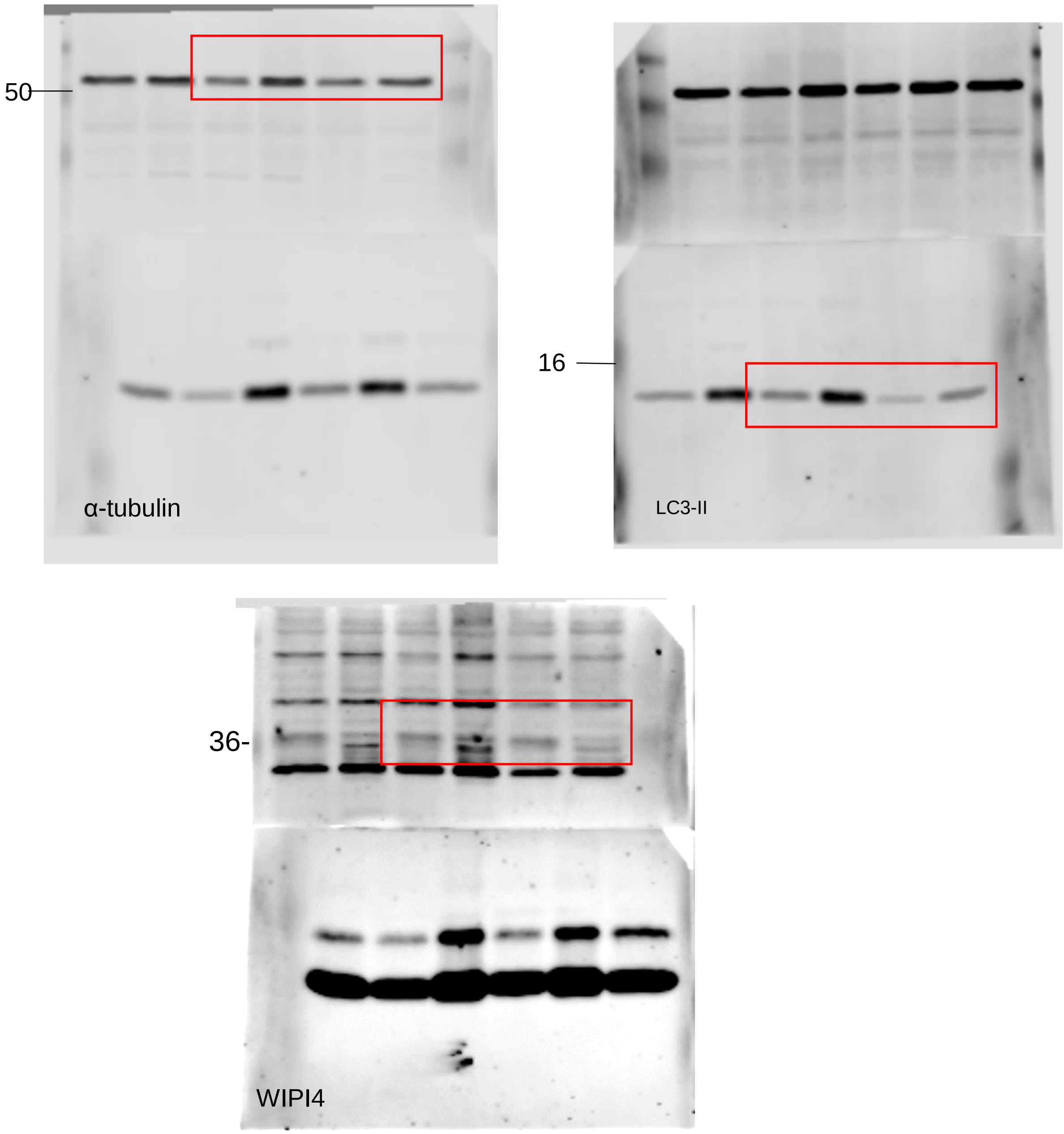

Figure S2g LC3-II levels in rapamycin treated HeLa cells

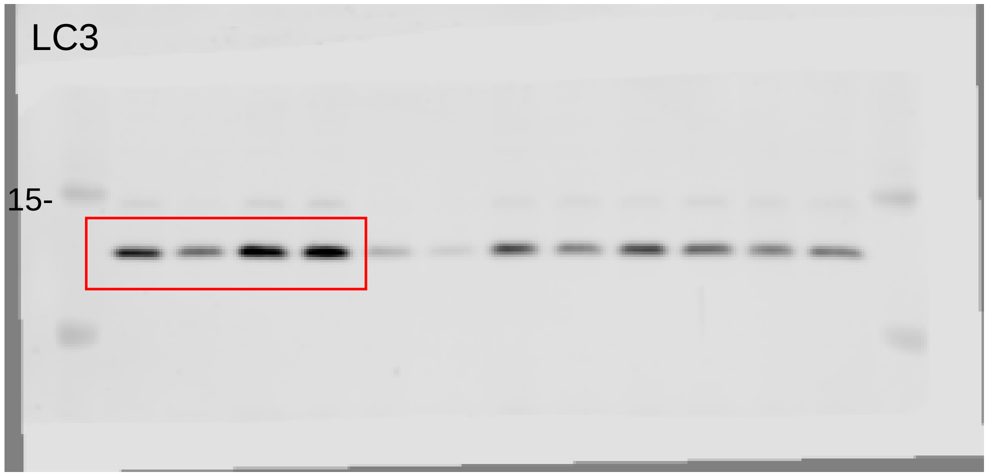

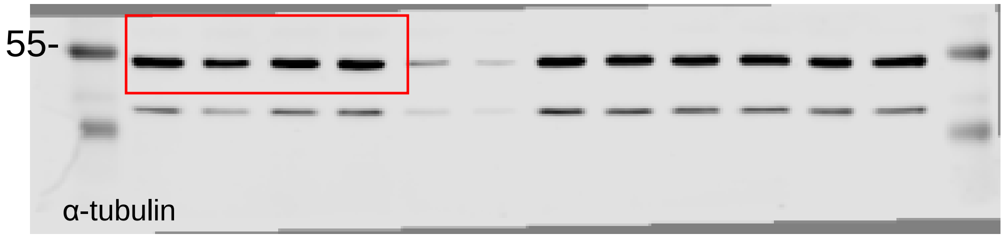

Figure S3a WIPI4 KD in ATG2A/B KO HeLa cells

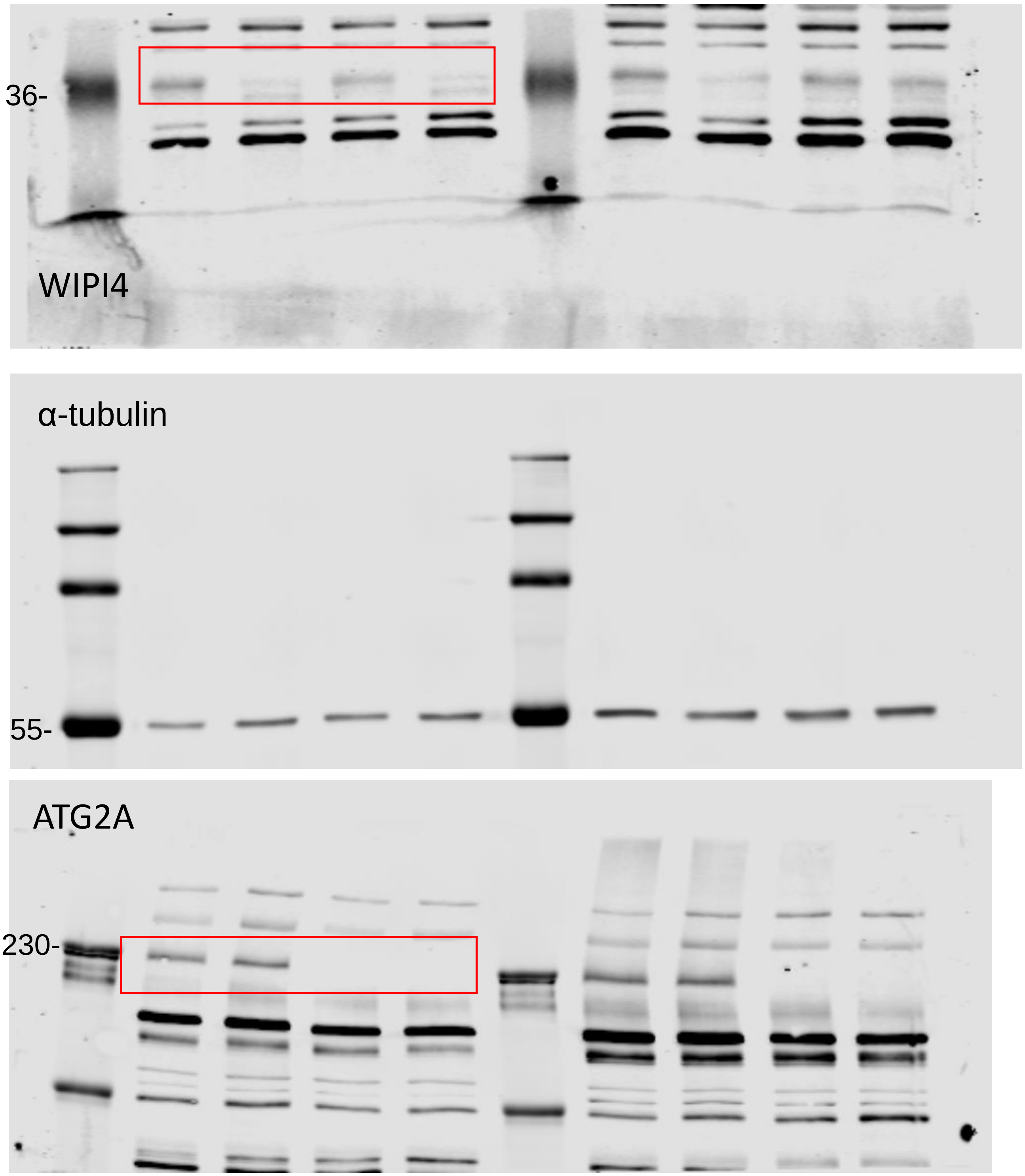

Figure S3d ATG2A and WIPI4 double KD efficiency in HeLa cells

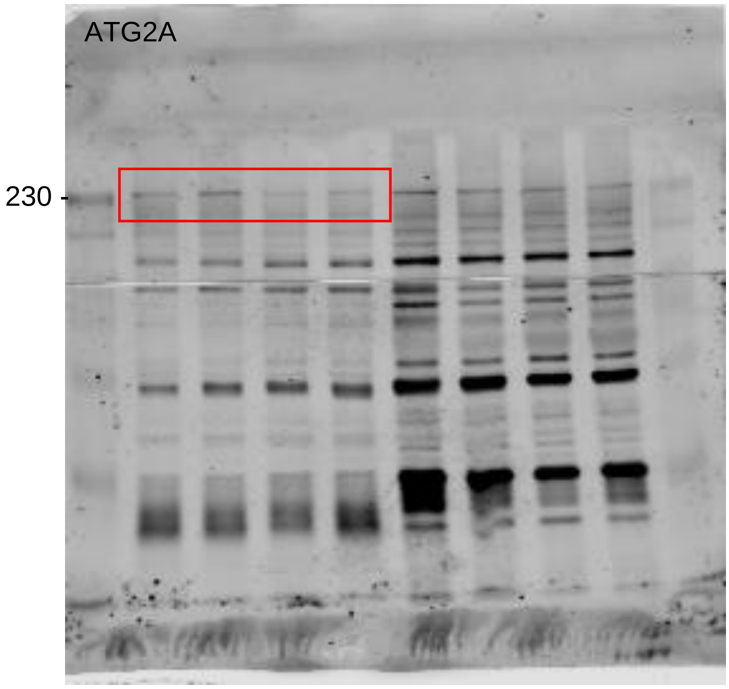

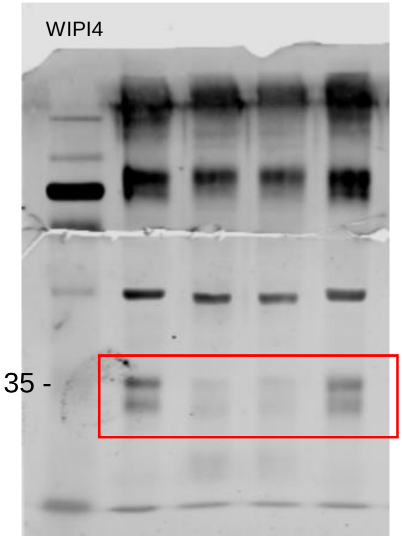

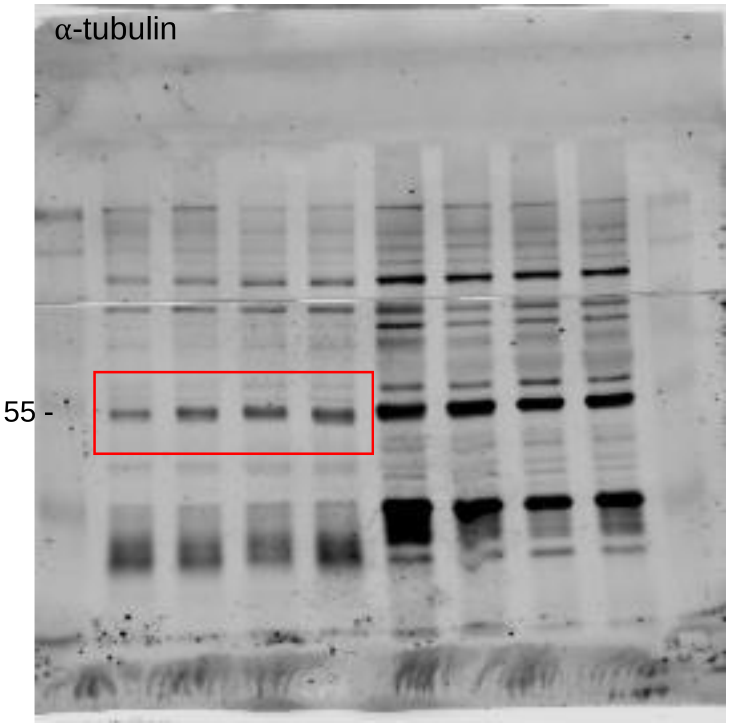

Figure S3e Expression levels of WIPI4-GFP and WIPI4 mutant

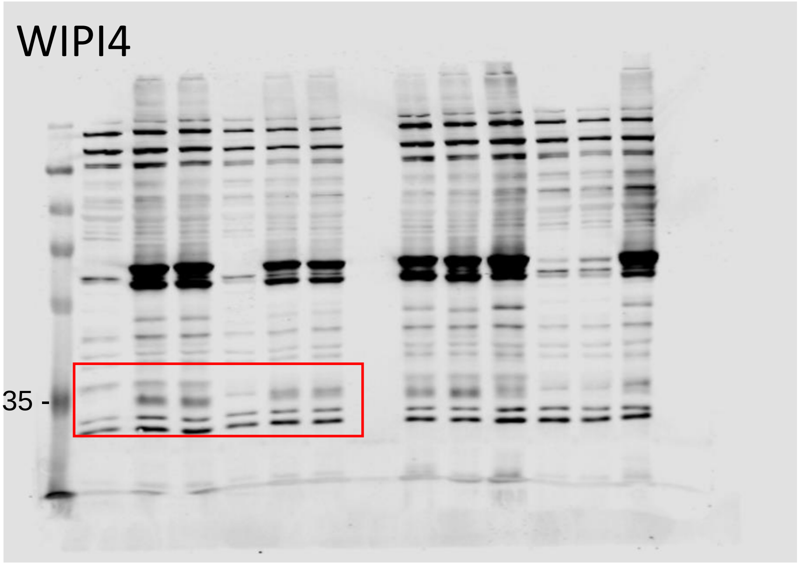

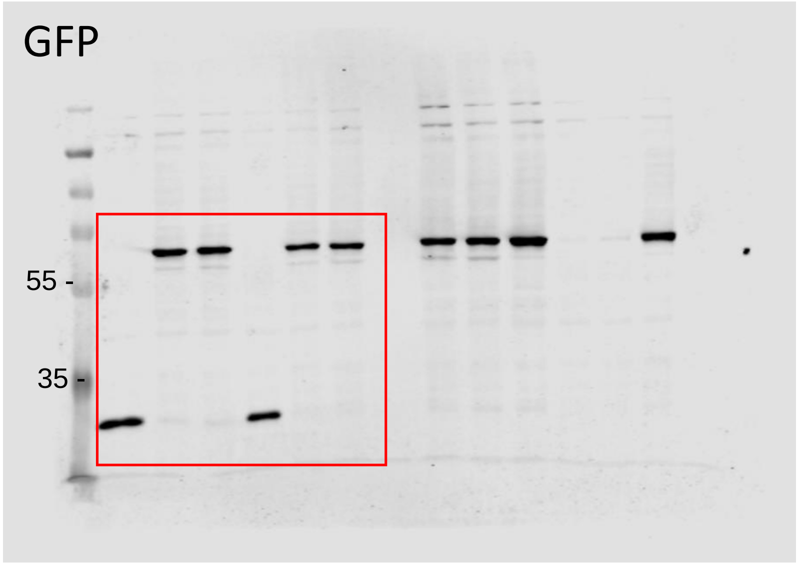

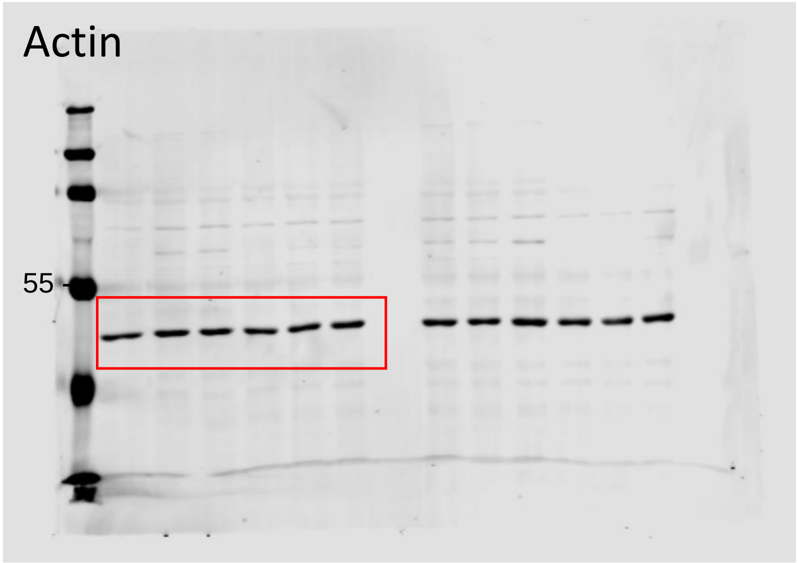

Figure S3f GFP trap to IP WIPI4 GFP and IB for ATG2A

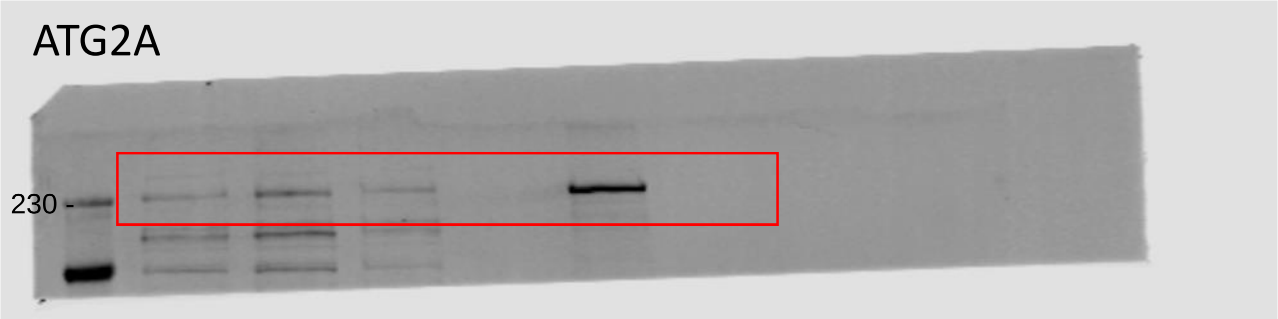

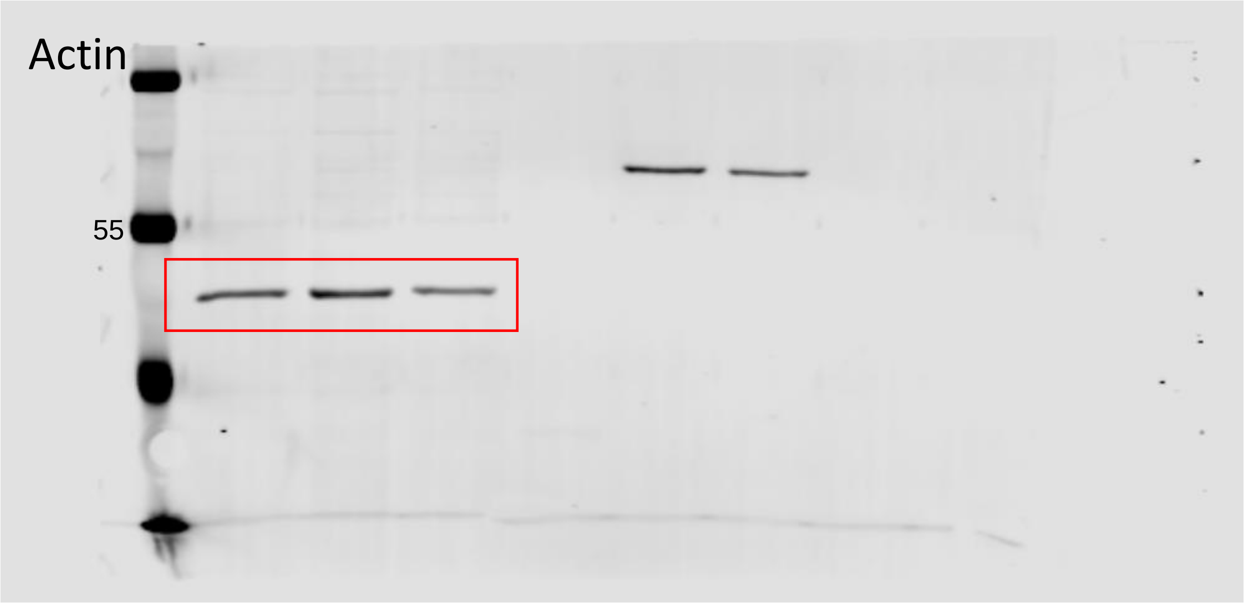

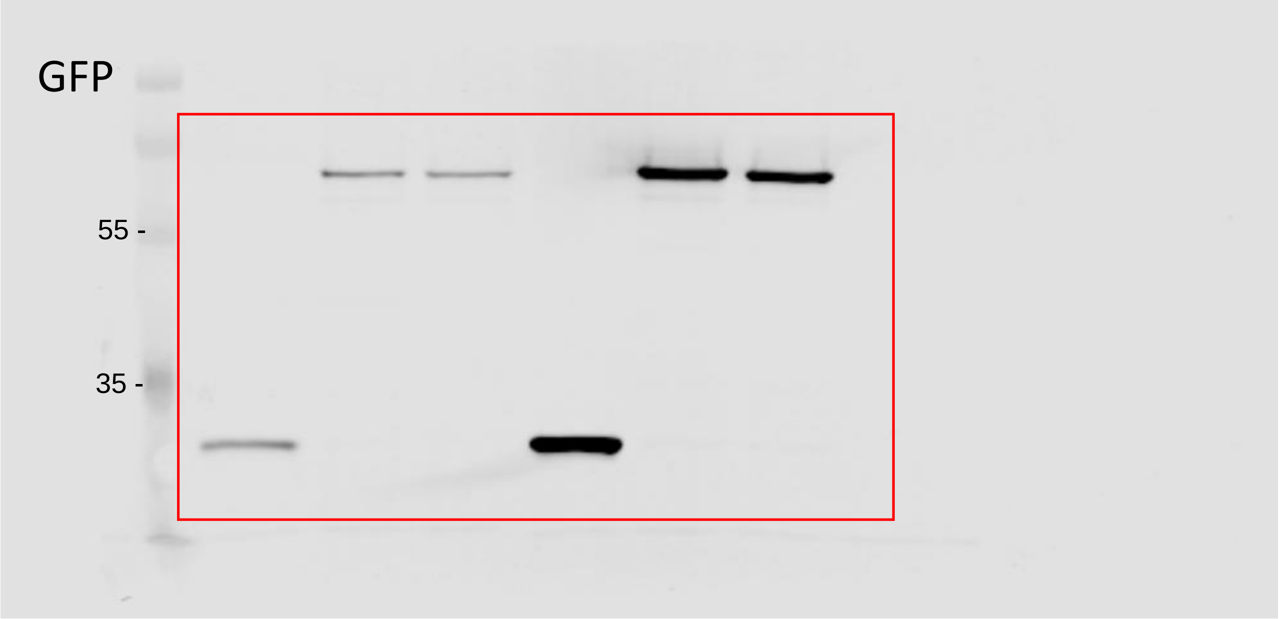

Figure S5a ATG2A IP pulled down ER and mitochondrial proteins

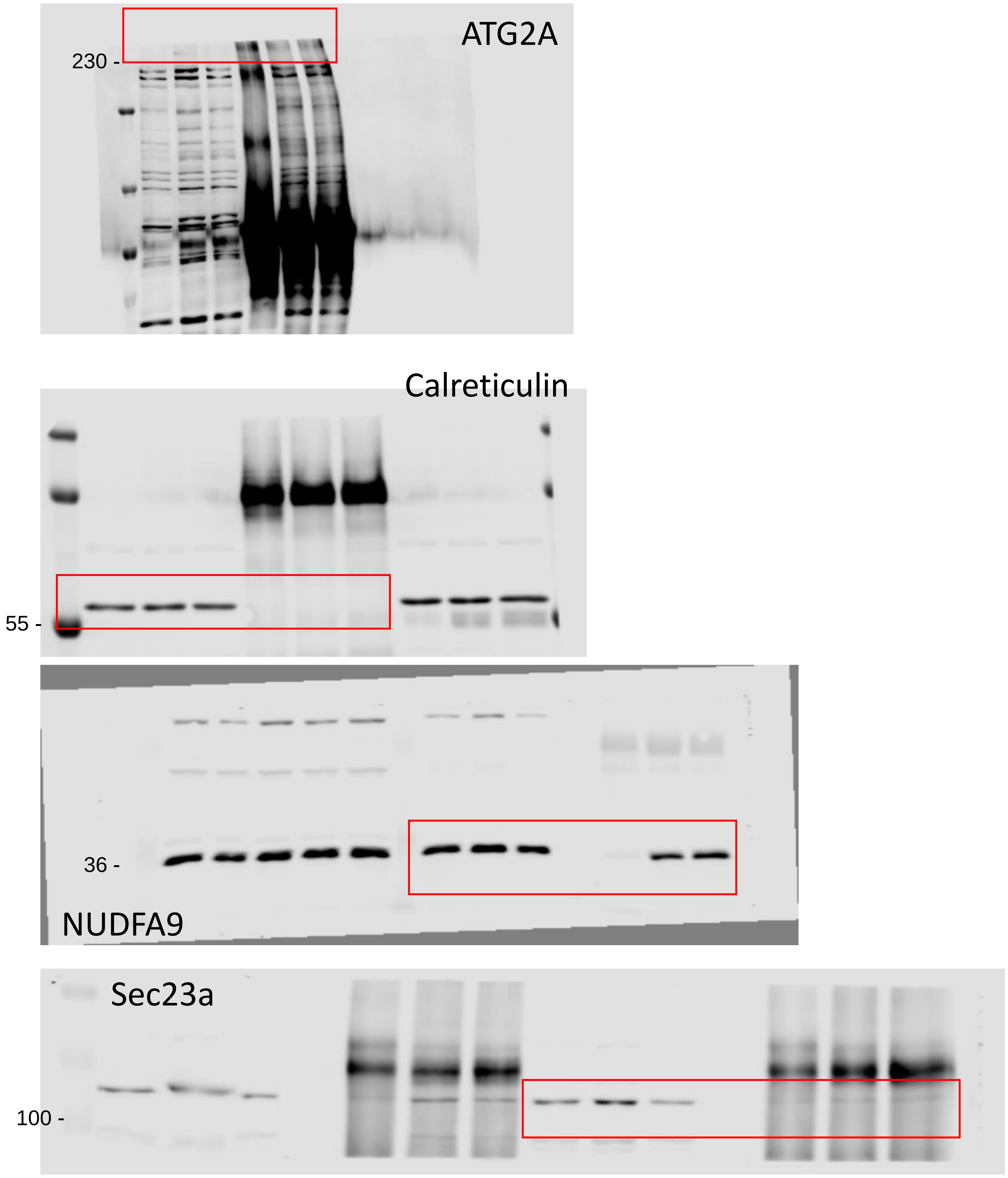

Figure S5a

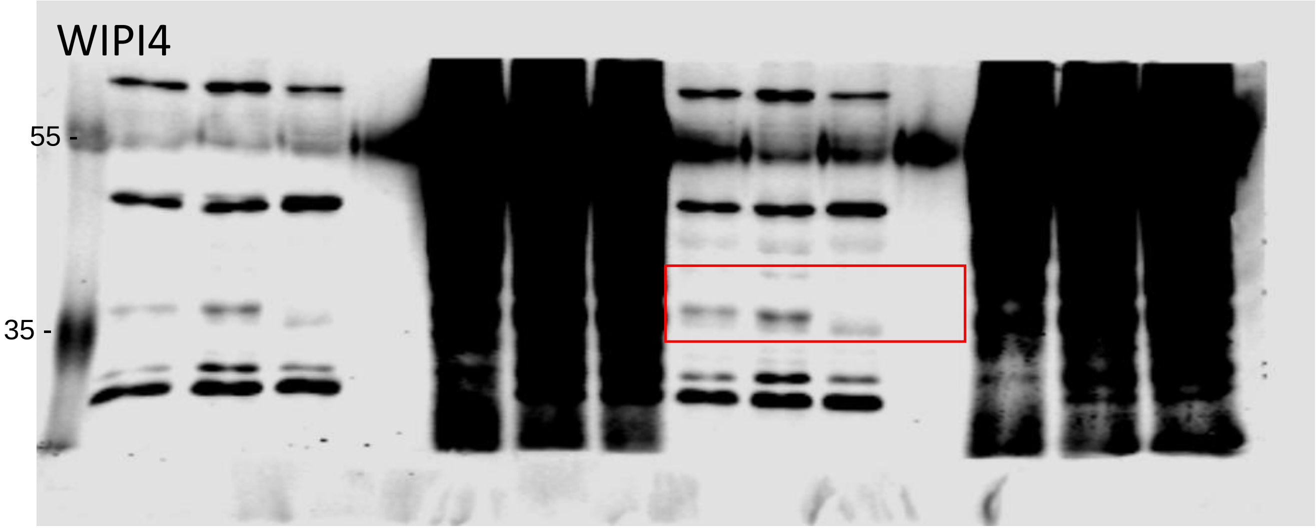

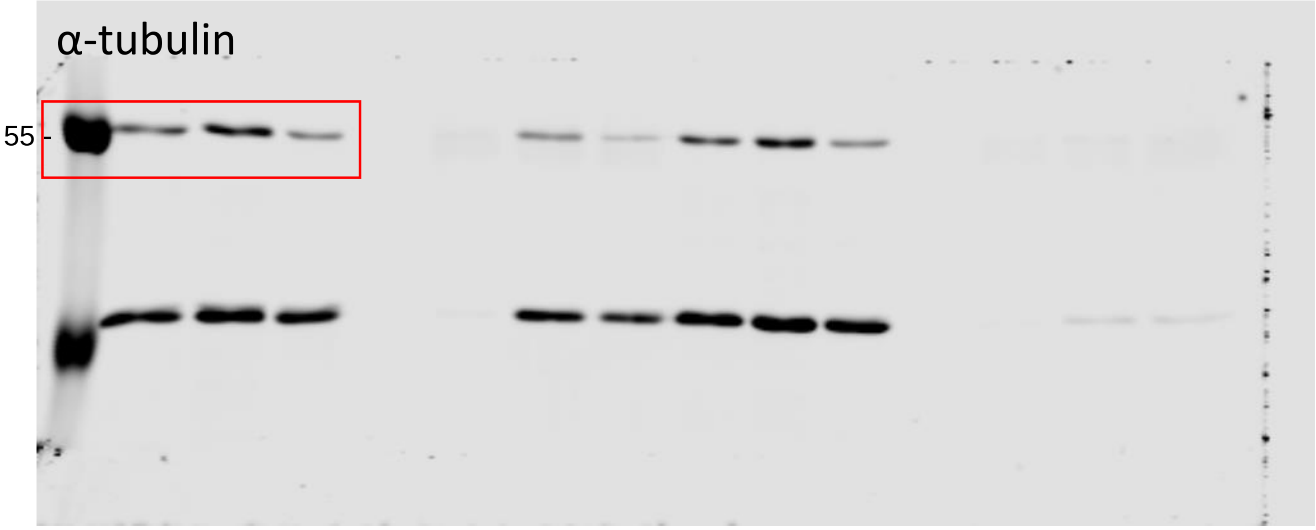

Figure S5c Expression levels of ATG2 GFP and ATG2mLIR mutant

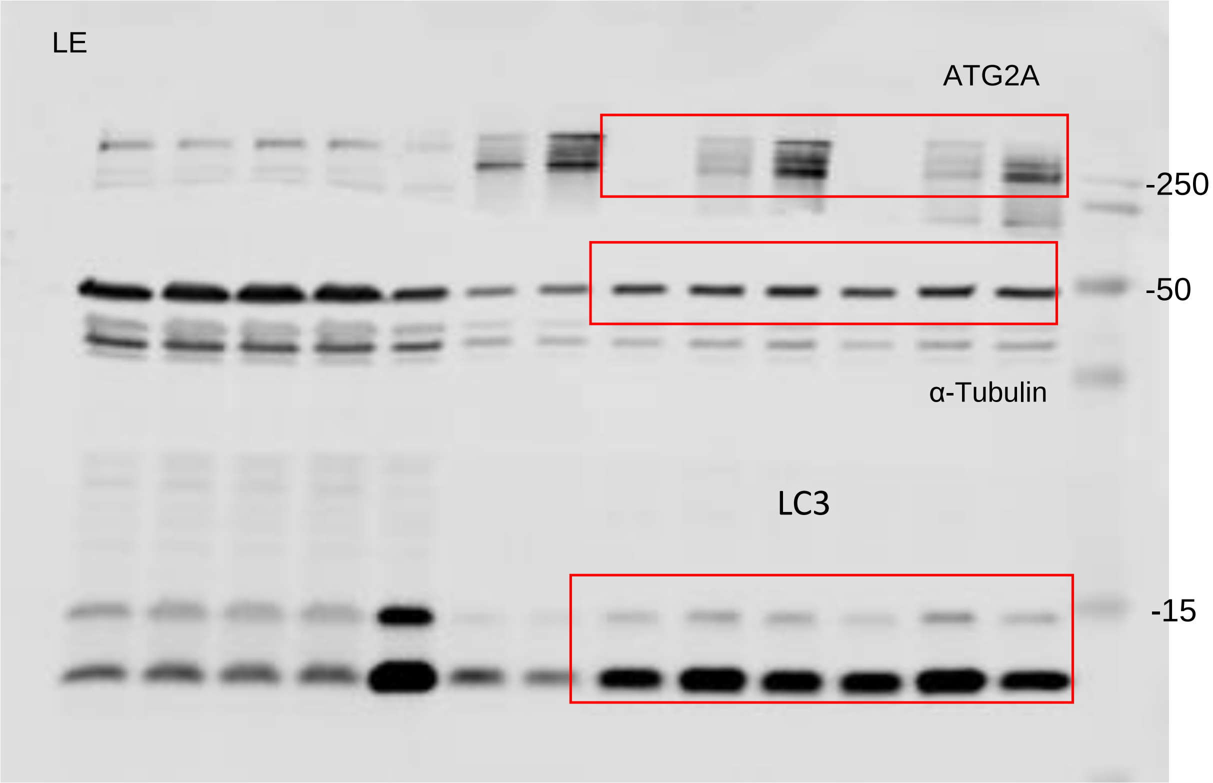

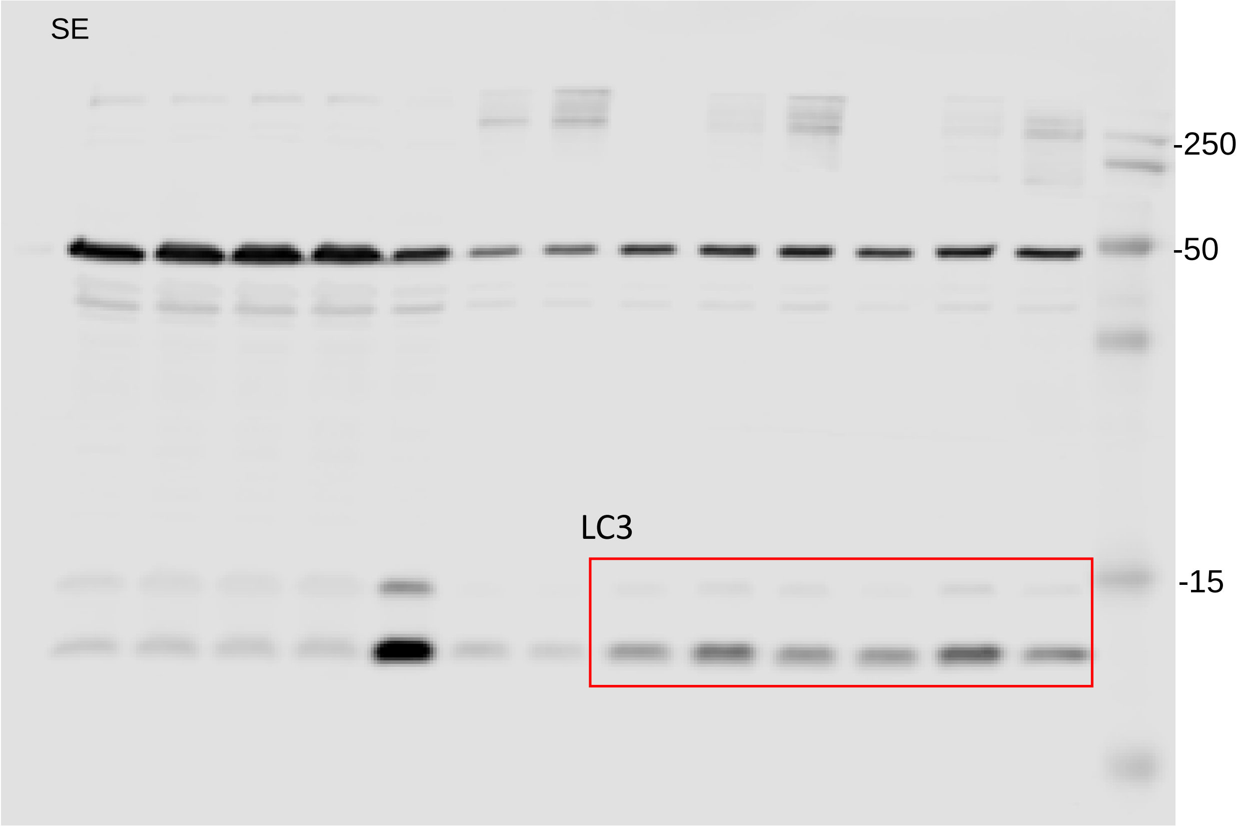

Figure S7a ATG2A IP pulled down both TOM40 and TMEM41B

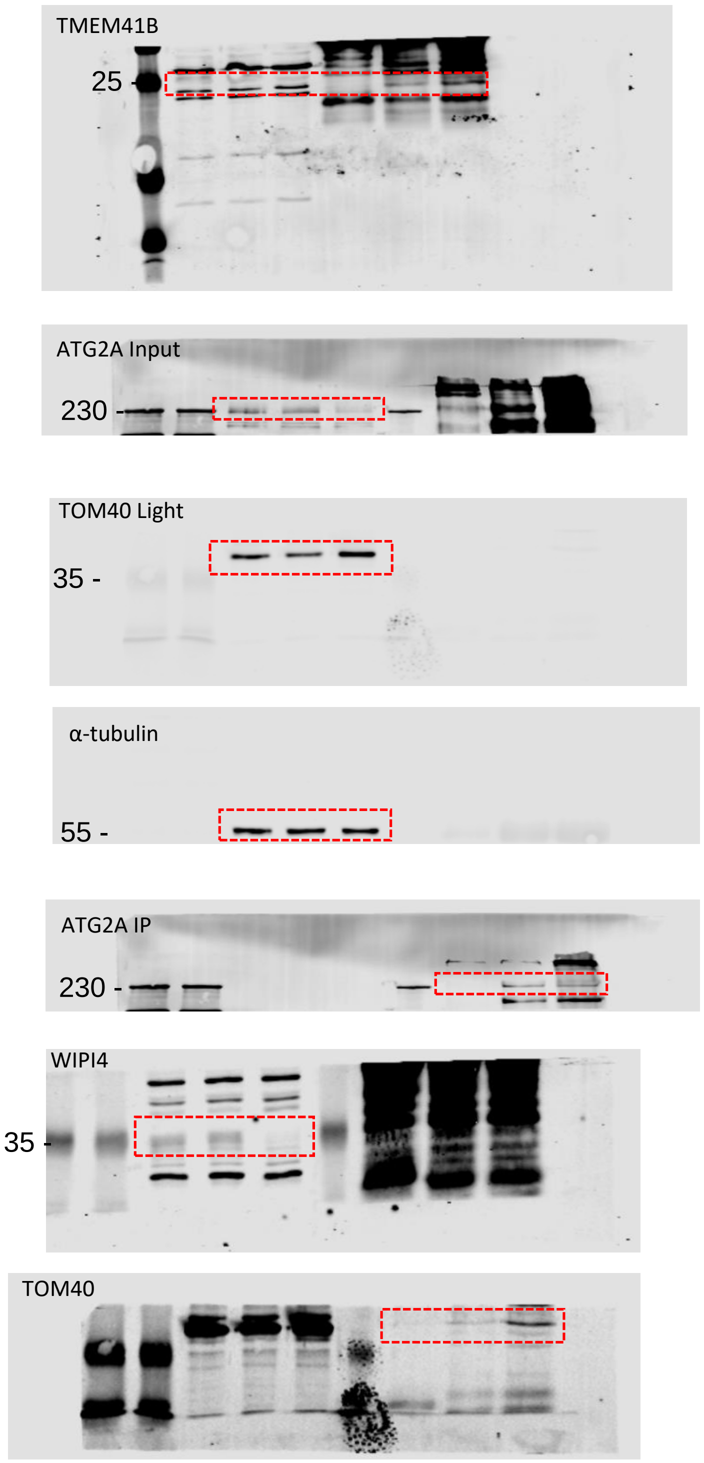

Figure S7e Day 15 iPSC neurons - differentiation levels

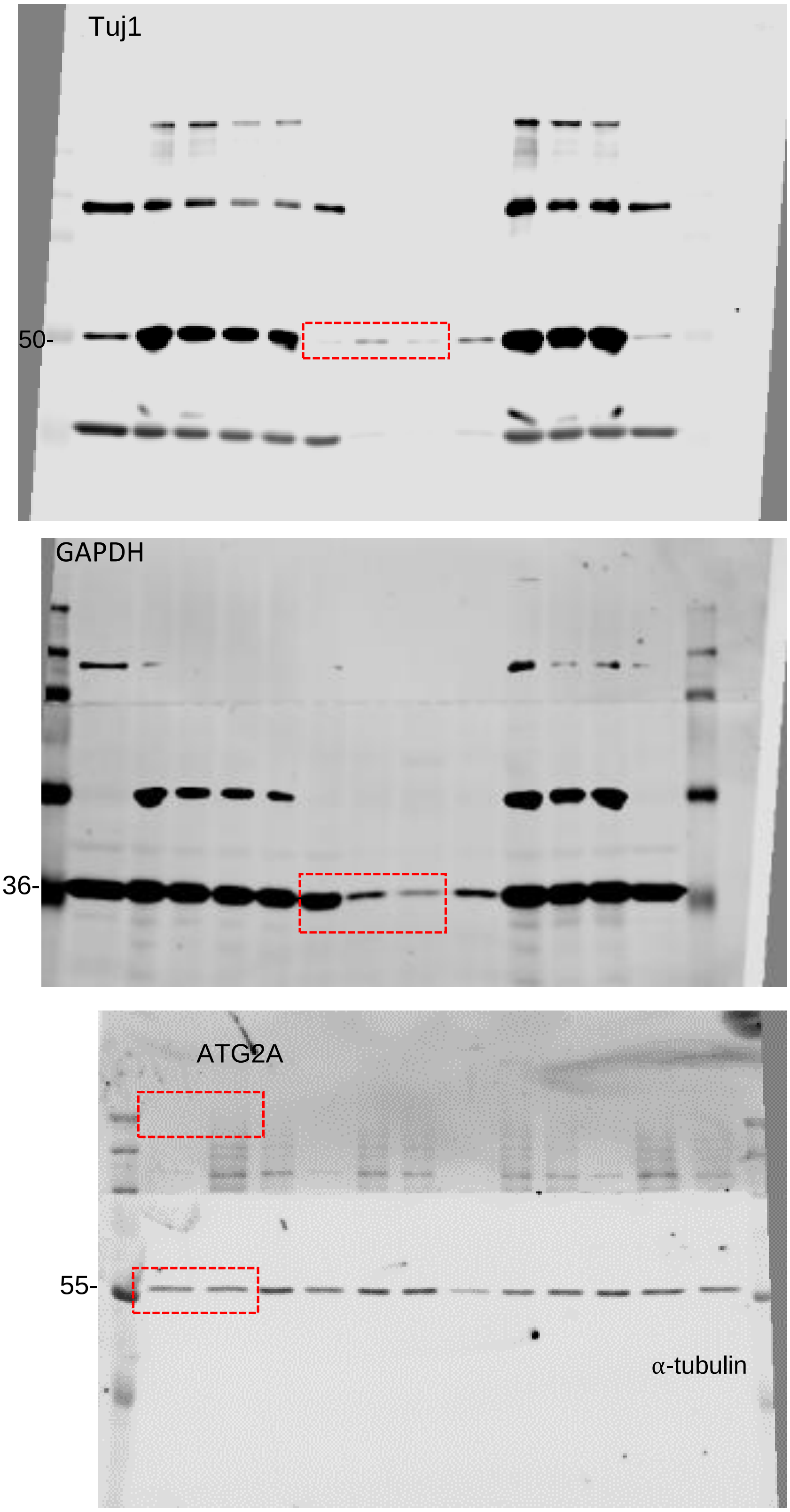

Figure S7f Mitochondria depletion efficiency in HeLa cells induced of mitophagy

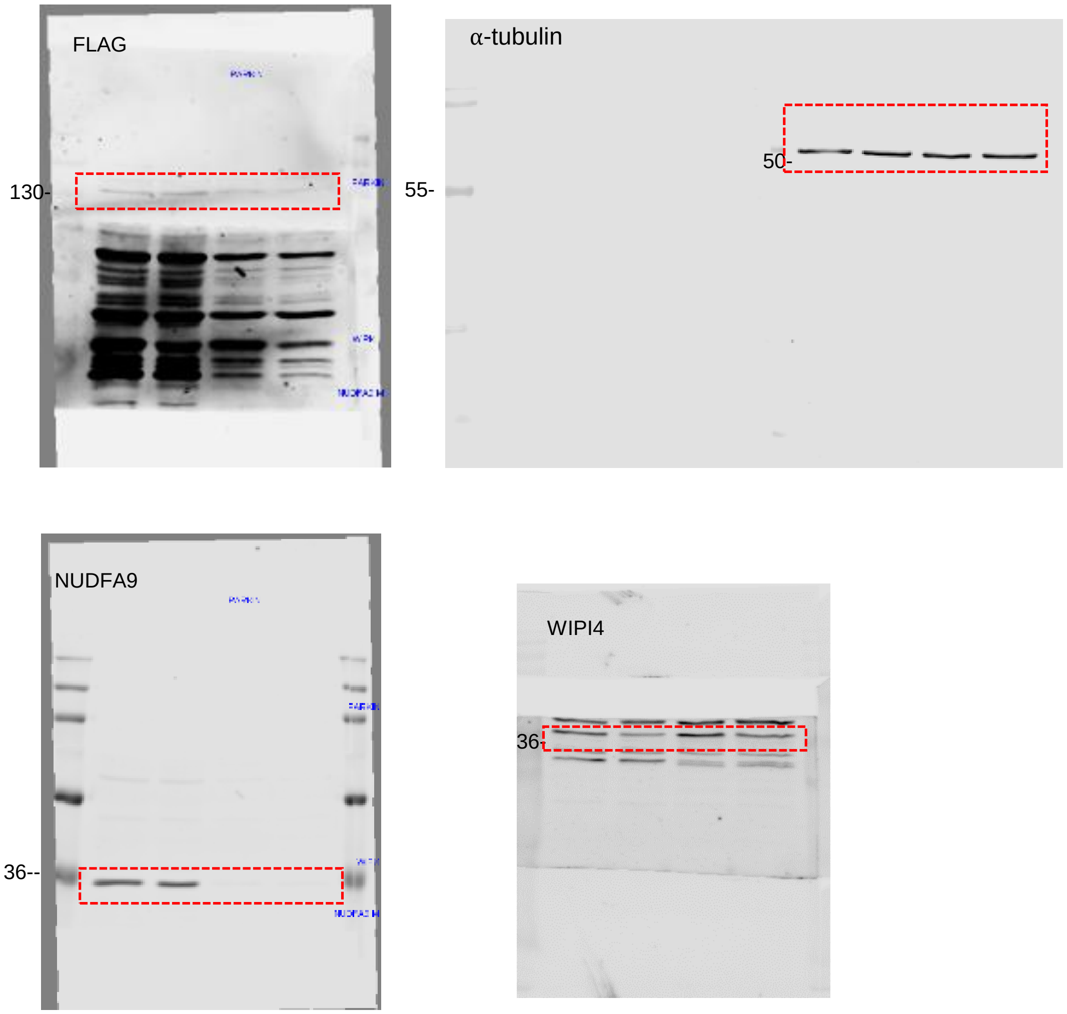

Figure S7g NIX and WIPI4 double KD efficiency

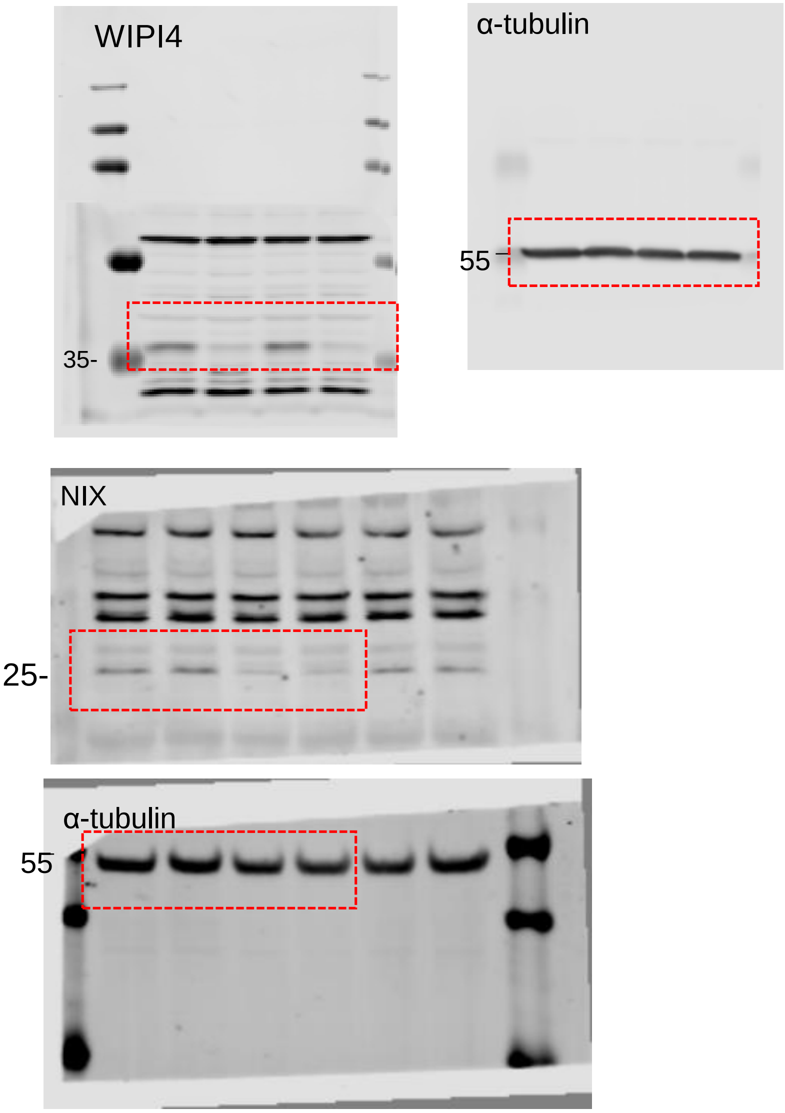

Figure S8a Expression levels of WT ATG2A GFP and ATG2A LTD GFP

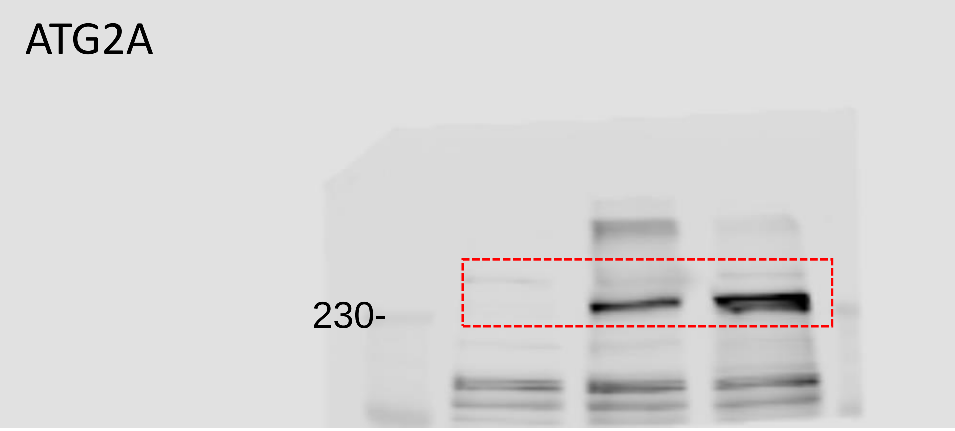

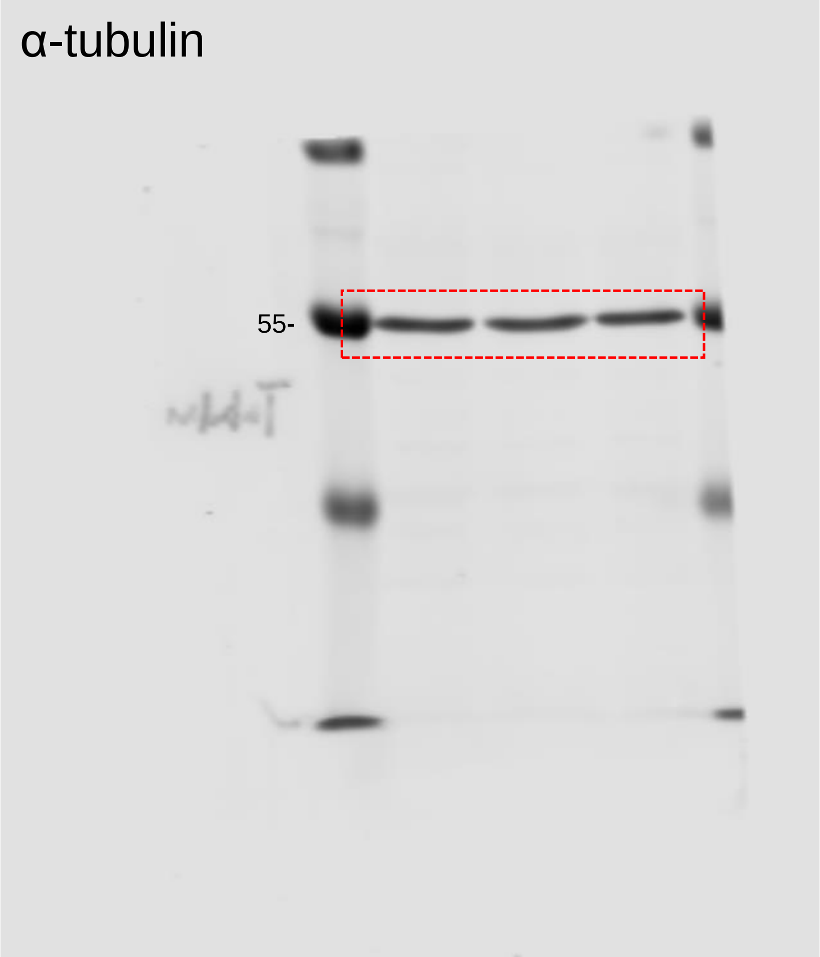

Figure S8d KD efficiency of WIPI4 by shRNA in iPS neurons and in primary neurons

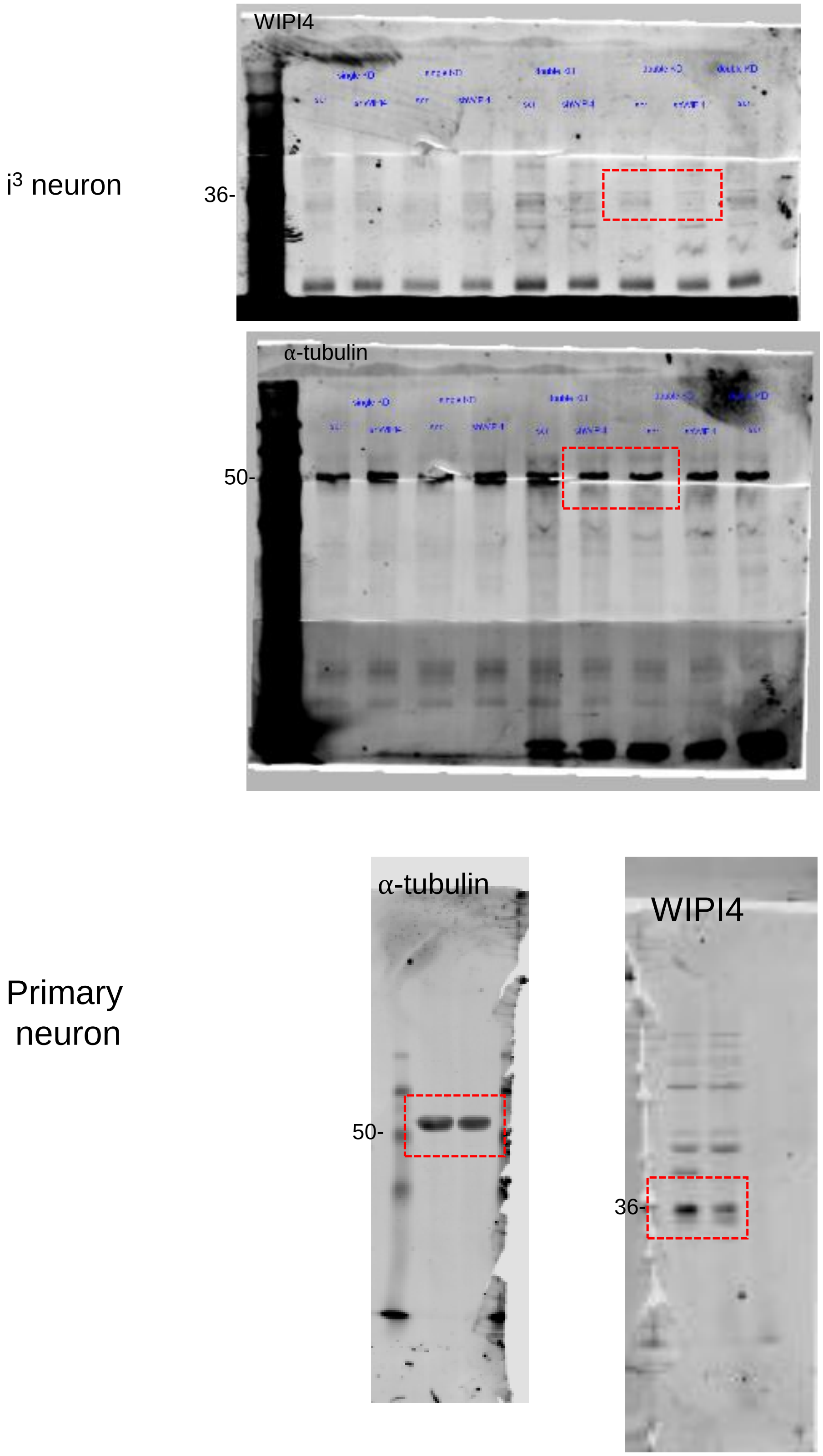

Figure S9a KD of PISD with two different siRNAs

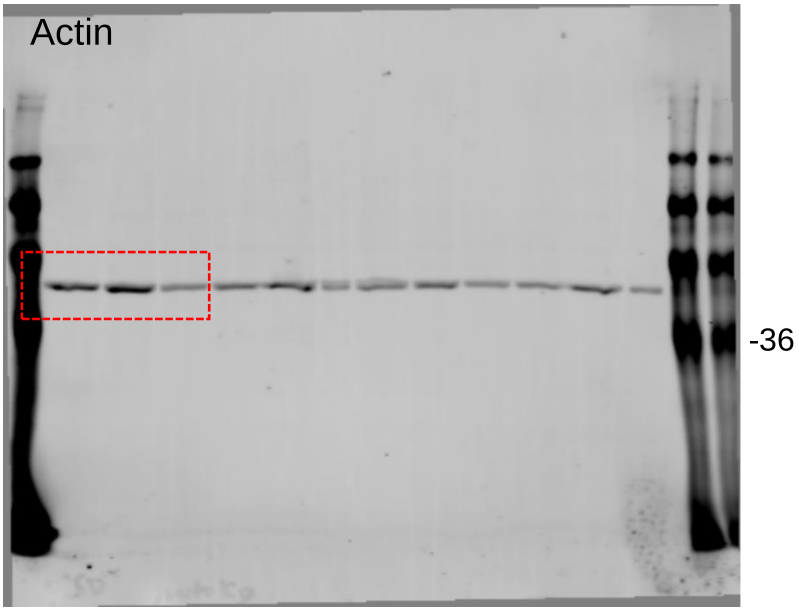

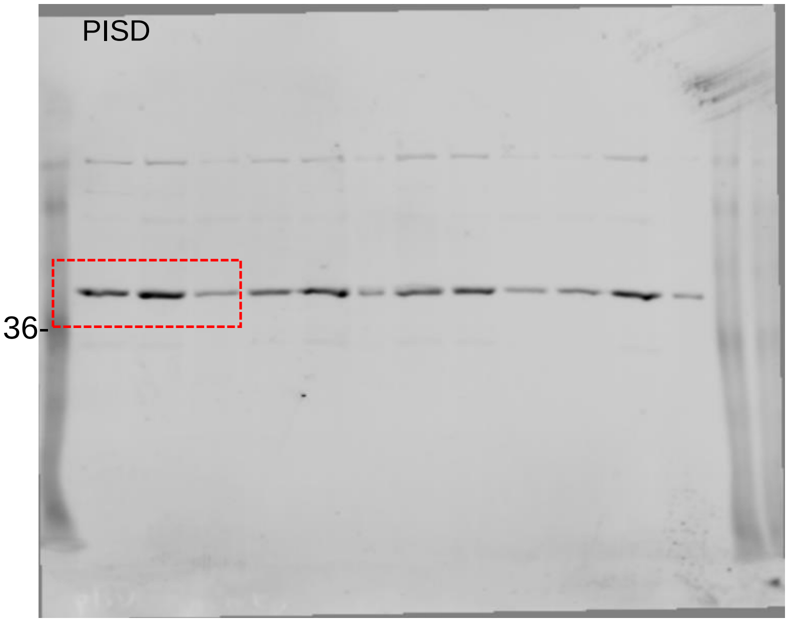

Figure S9b PISD levels in different cells fractions

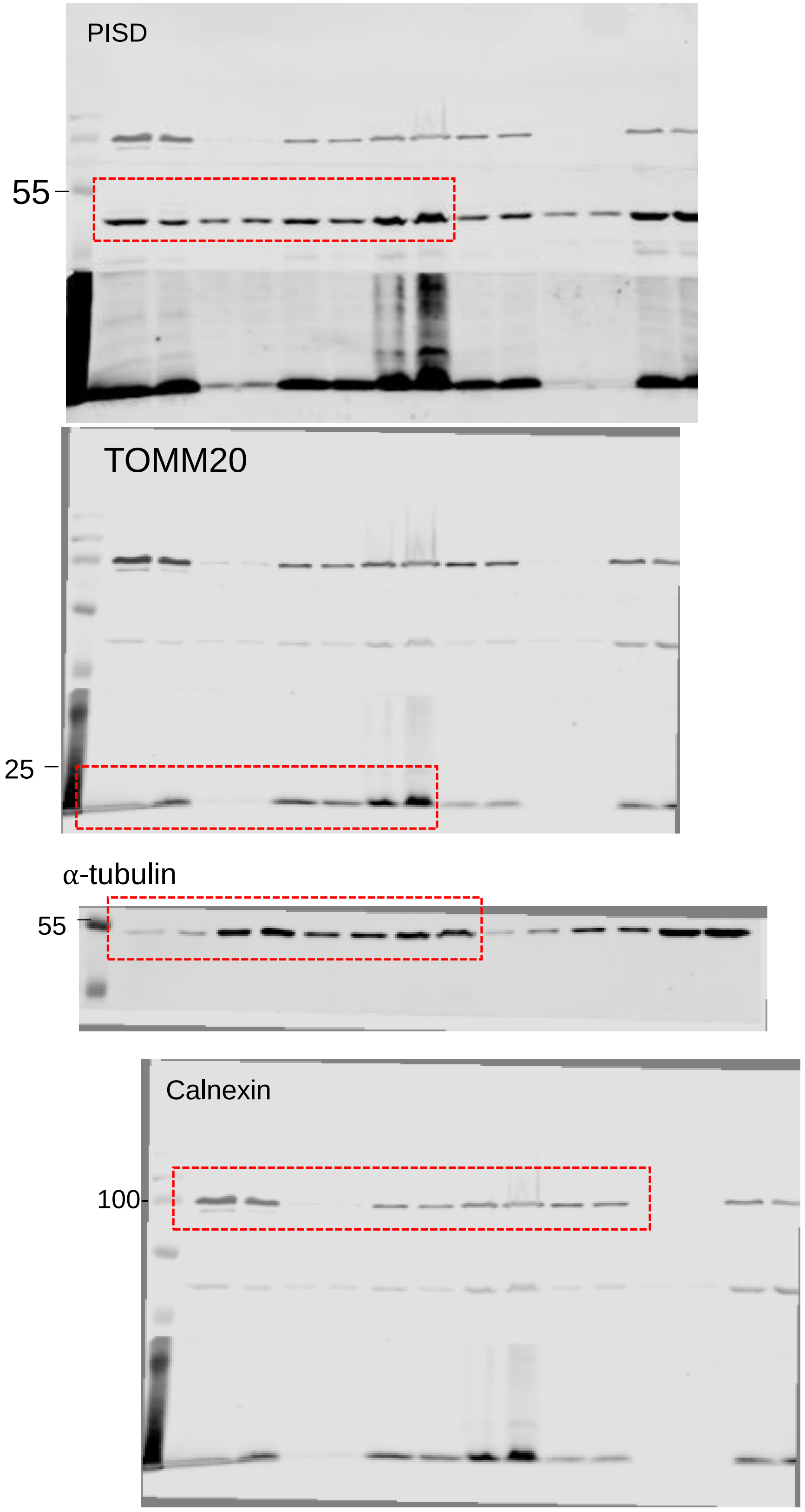

Figure S9c double KD efficiency of PISD and WIPI4

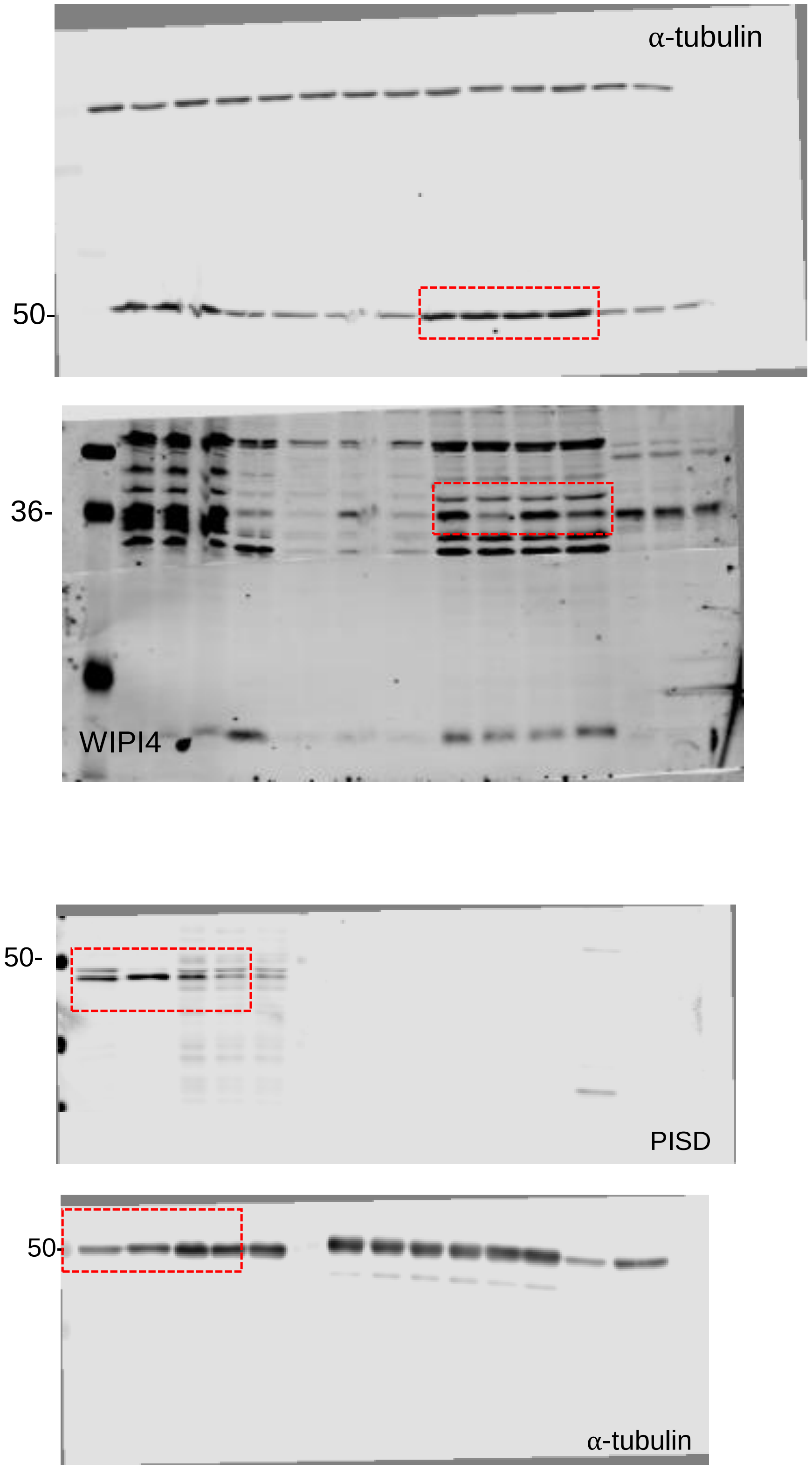

Figure S9d double KD efficiency of PISD and WIPI4

PISD

50-

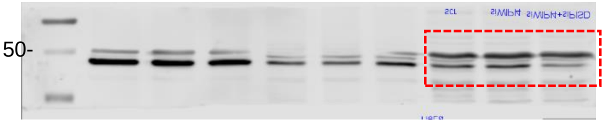

α-tubulin

50-

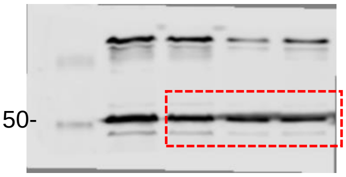

WIPI4

36-

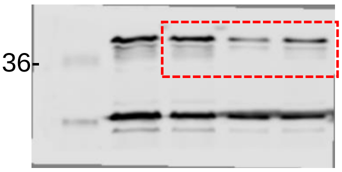

Figure S9e Overexpression efficiency of PISD-FLAG and it empty control

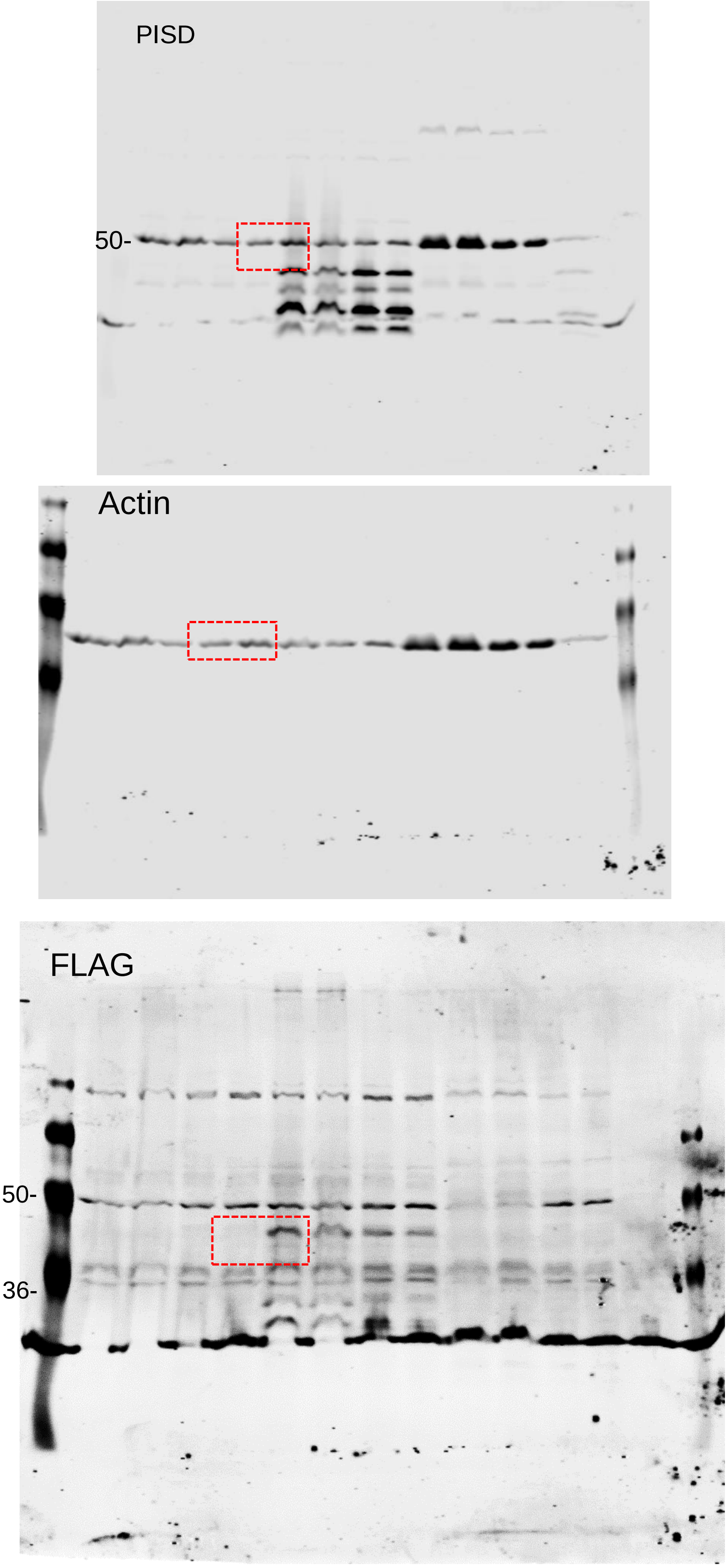

Figure S10b Triple knockdown efficiency of WIPI4, ORP5 and ORP8

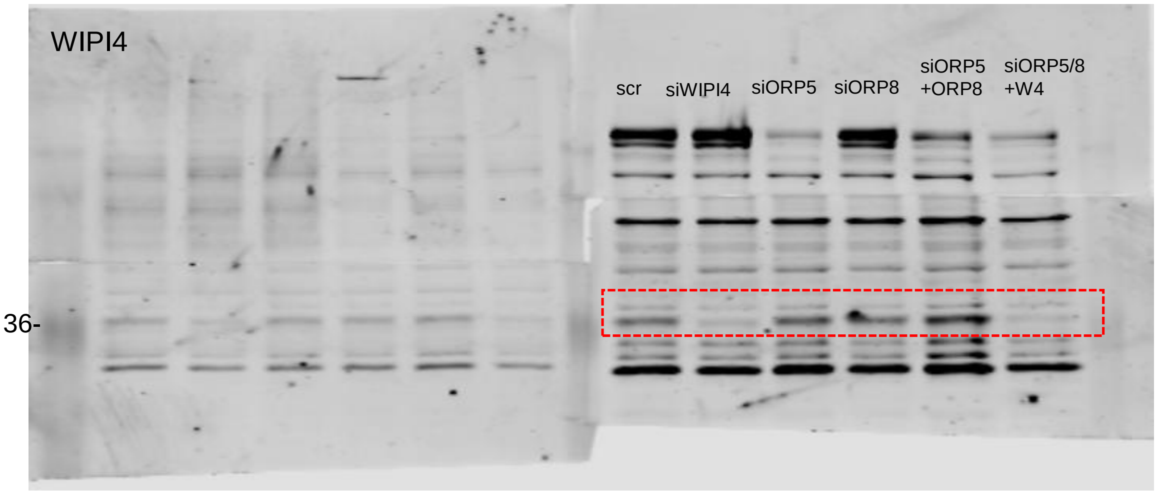

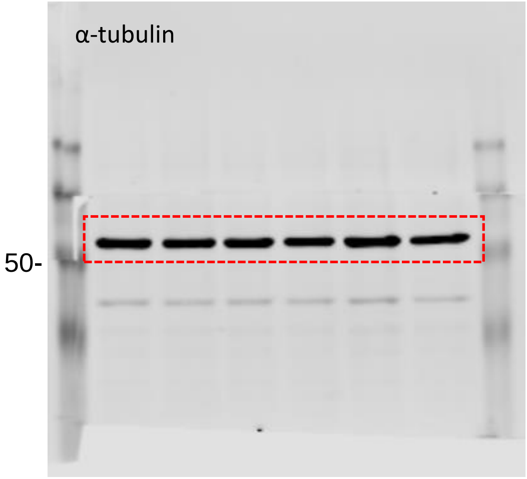

Figure S10c

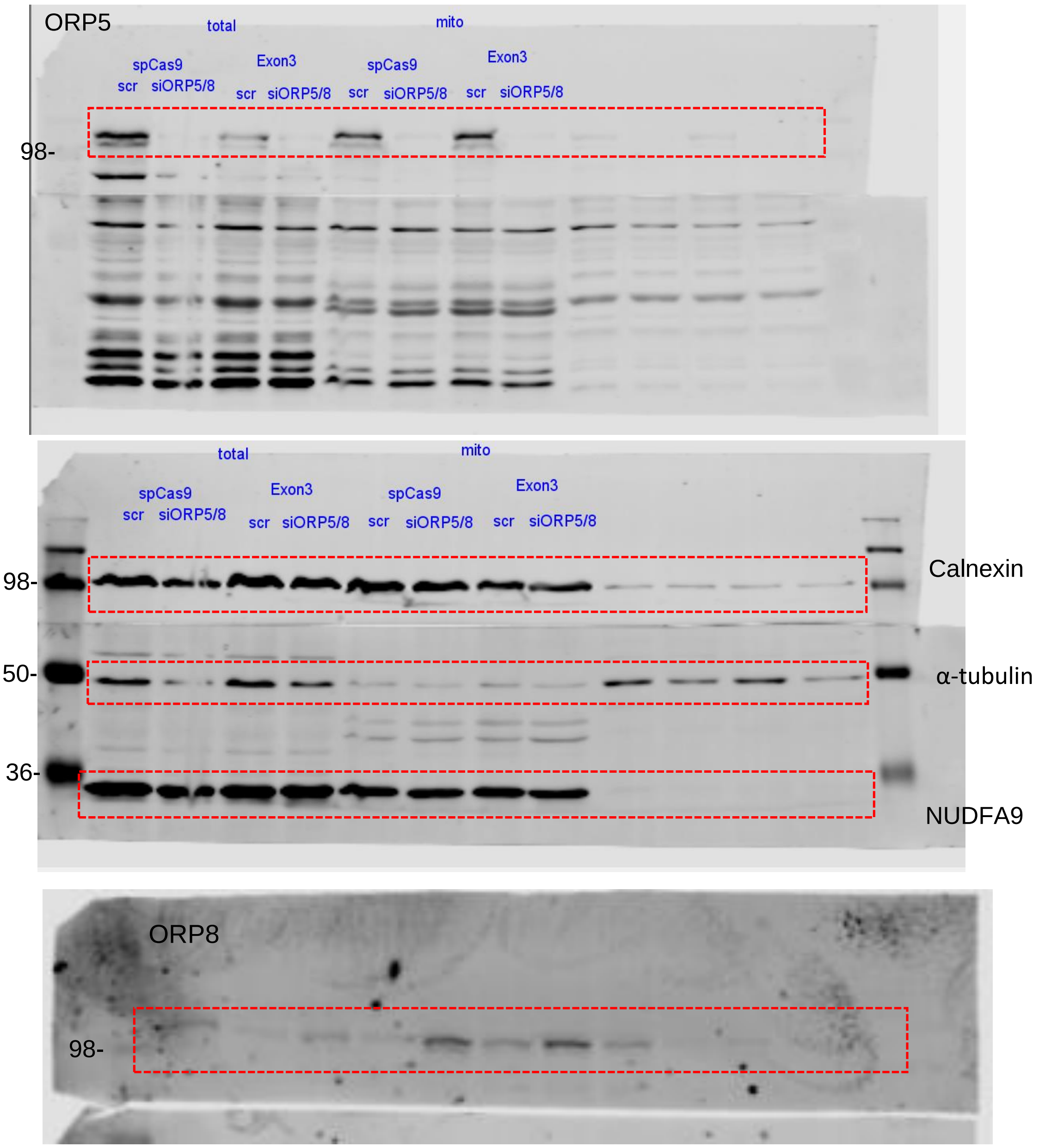
ORP5
total
mito
spCas9
scr
siORP5/8
Exon3
scr
siORP5/8
spCas9
scr
siORP5/8
Exon3
scr
siORP5/8
98-
total
mito
spCas9
scr
siORP5/8
Exon3
scr
siORP5/8
spCas9
scr
siORP5/8
Exon3
scr
siORP5/8
98-
50-
36-
Calnexin
α-tubulin
NUDFA9
ORP8
98-
